# Supplementary material for: Tau affects P53 function and cell fate during the DNA damage response
Source: Commun Biol. 2020 May 19;3:245. doi: 10.1038/s42003-020-0975-4 (PMC7237658; doi:10.1038/s42003-020-0975-4)
Supplement: Supplementary file 1 — Supplementary Information [file 42003_2020_975_MOESM1_ESM.pdf]

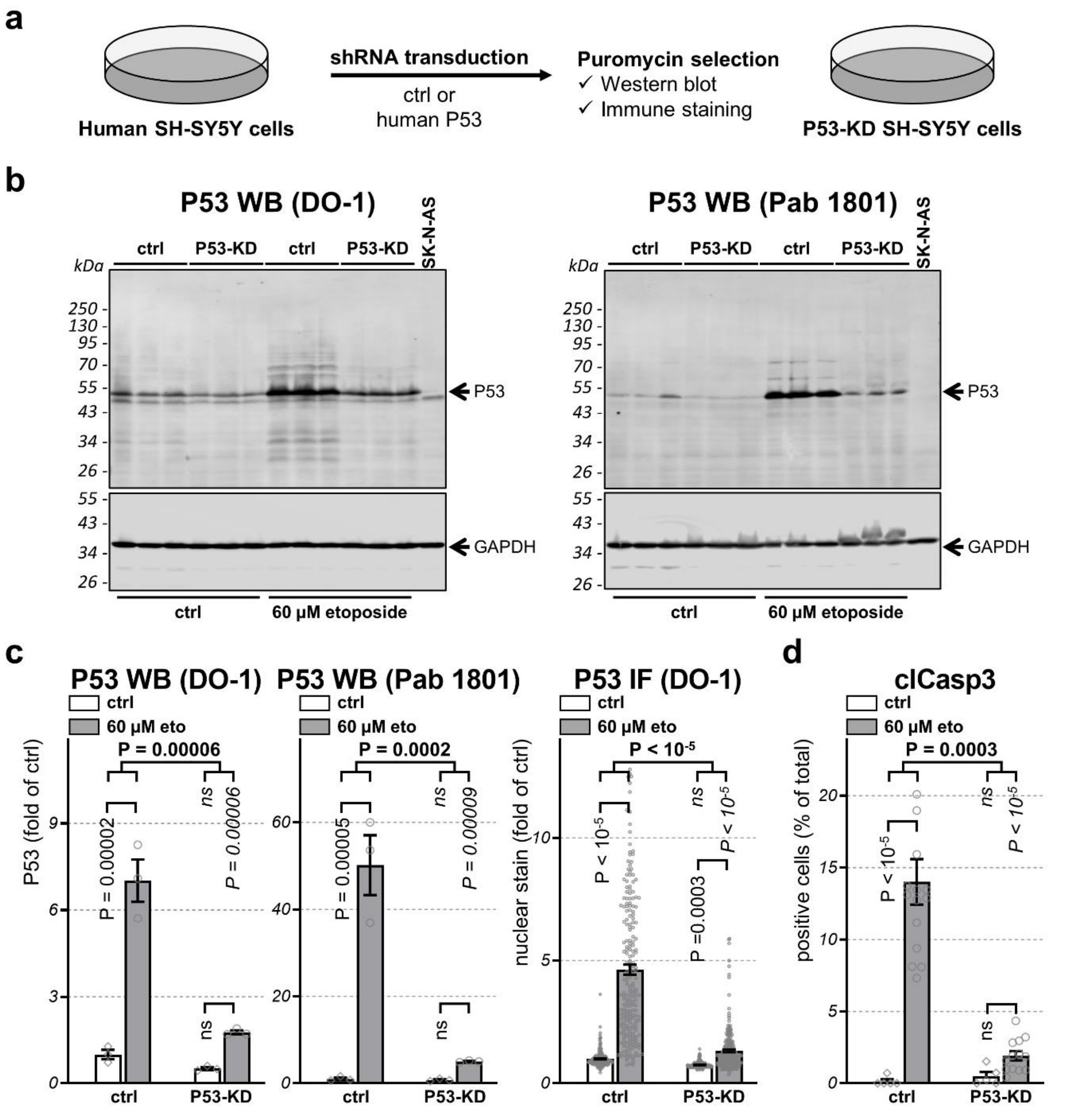

Supplementary Figure 1. DSBs-induced apoptosis is P53-dependent in SH-SY5Y cells. a. Scheme of procedure used to generate P53-KD cells and for their characterization by western blot and P53 immune staining when compared to cells transduced with the parental shRNA plasmid (ctrl). **b.** Cells at basal conditions (ctrl) or treated 30 min with 60 μM etoposide and recovered for 6 h before western blot analysis with the indicated P53 antibodies. GAPDH served as loading control. **c.** Western blot quantification of P53 signal intensity normalized for GAPDH shown as fold of control cells at basal conditions, mean +/- SD of n = 3 biological replicates. On the left is show the quantification of P53 immune staining as fold of control cells at basal conditions, mean

intensity +/- sem of single cell nuclear P53 staining (DAPI mask, ImageJ), n >219 cells/condition distributed over 5 images. **d**. Quantification of clCasp3 immune staining shown as percent of total DAPI-positive cells, mean ± SD of 5 images for the untreated cells (ctrl) and of 15 images for etoposide-treated cells (60 µM eto), n >500 cells/condition. Statistical analysis by independent measures ordinary 2way ANOVA, source of variation for cell lines (in bold), multiple Bonferroni pairwise comparisons among lines for each treatment (in italics), or among treatment for each line (in vertical).

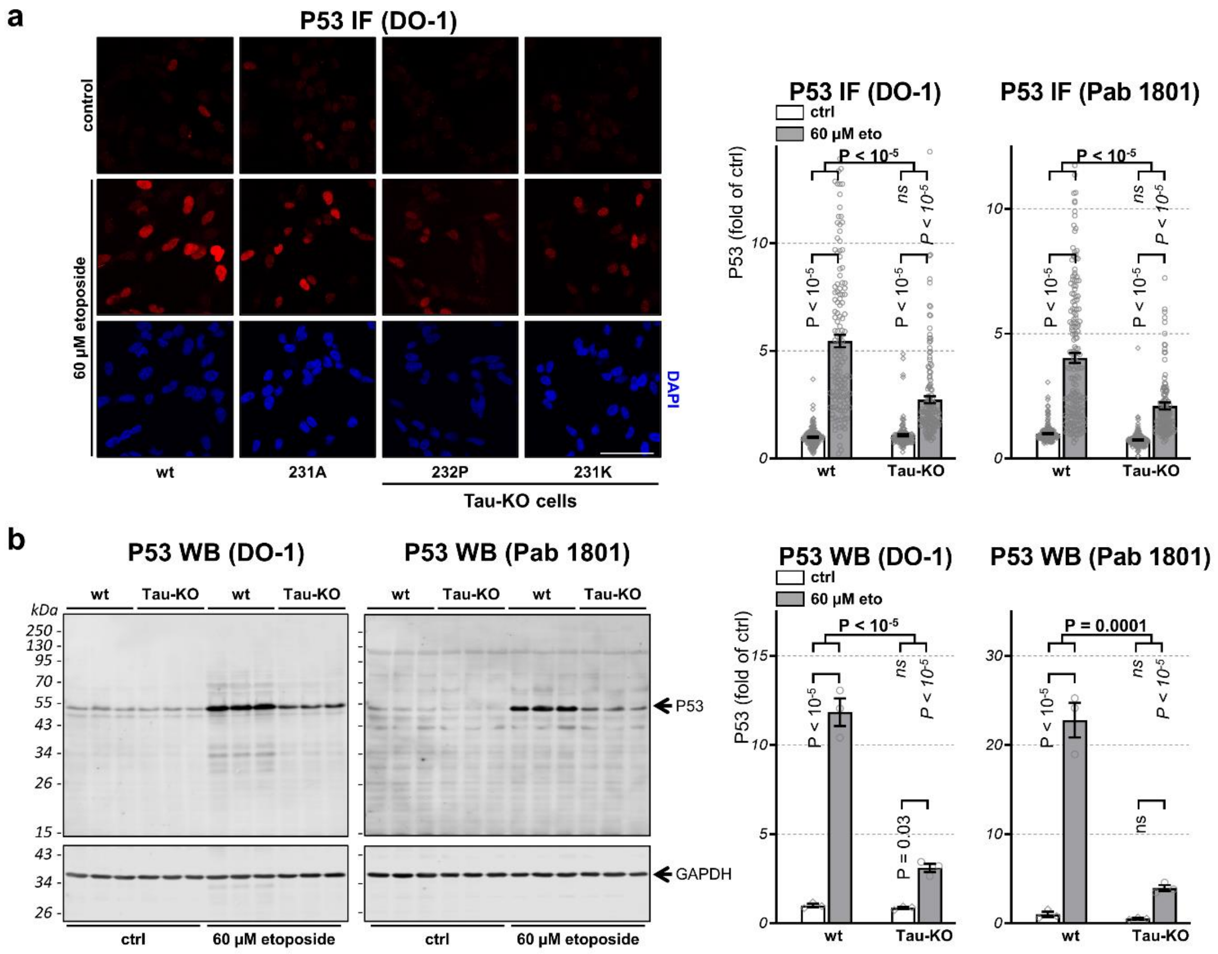

Supplementary Figure 2. Tau deficiency reduces cellular P53. a. Representative laser confocal microscopy images of P53 DO-1 antibody-immune stained parental cells (wt) and the indicated CRISPR-Cas9 cell lines at basal conditions (control) or treated for 30 min with 60 µM etoposide and recovered for 6 h. Shown are also the nuclear staining with DAPI. Scale bar 50 µm. Quantification of mean intensity +/- sem of single cell nuclear P53 staining (DAPI mask, ImageJ) shown as fold of wt cells at basal conditions using the indicated P53 antibodies, n >90 cells/condition distributed over 5 images. **b.** Parental (wt)

or 232P (Tau-KO) cells at basal conditions (ctrl) or treated for 30 min with 60 μM etoposide followed by 6 h recovery were analyzed for P53 by western blot with the indicated P53 antibodies, n = 3 biological replicates. GAPDH served as loading control. Quantification of P53 signal intensity normalized for GAPDH, mean +/- SD shown as fold of control (wt or ctrl cells at basal conditions). Statistical analysis by independent measures ordinary 2way ANOVA, source of variation for cell lines (in bold), multiple Bonferroni pairwise comparisons for each treatment among lines (in italics), or among treatment for each line (in vertical).

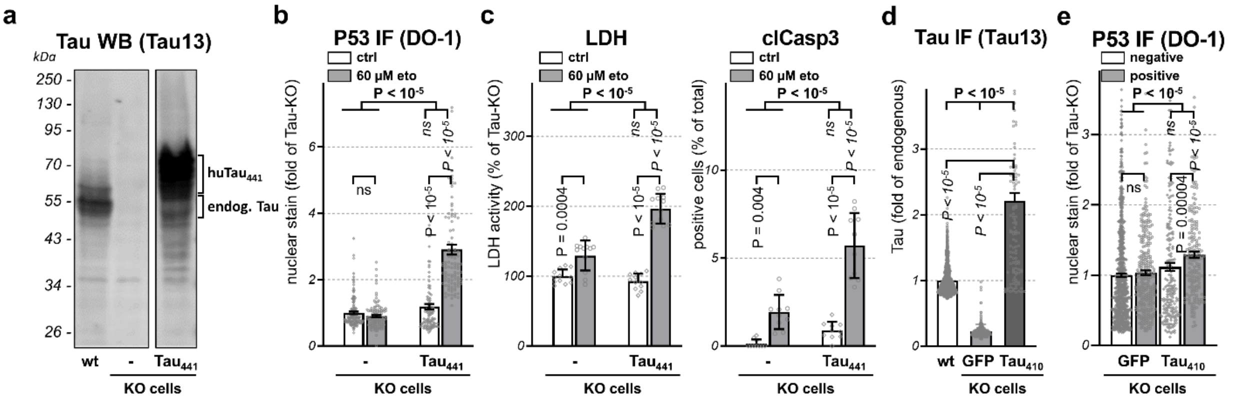

Supplementary Figure 3. Restoring Tau expression in Tau-KO cells rescues P53 and the apoptotic phenotype. a. Parental (wt), 232P (KO cells) or 232P cells stably re-expressing 4R-Tau (Tau_{441}) analyzed by western blot with the Tau13 antibody. **b.** Quantification of single cell nuclear P53 immune staining (DAPI mask, Image J) of 232P Tau-KO cells or 232P-Tau_{441} cells at basal conditions (ctrl) or treated 30 min with 60 μM etoposide and allowed to recover 6 h (60 μM eto), mean ± sem of n >100 cells/condition distributed over 5 images shown as fold of 232P cells at basal conditions. **c.** LDH release from the same cells treated as in **b.** (2 d recovery). Values are shown as percentage of Tau-KO cells at basal conditions, mean ± SD of 12 wells from three independent experiments. Cells were also analyzed by immune staining for clCasp3 at 6 h recovery. Percent clCasp3-positive cells, mean ± SD of 10 images, n >500 cells/condition. **d.** Quantification of Tau13 immune stained Tau in parental cells (endogenous Tau, wt), or in GFP-positive 232P cells (KO cells) transiently transfected with a 1:10 ratio of empty:GFP plasmids (GFP) or of 3R-Tau:GFP plasmids (Tau_{410}). Cells were stained 3 days after transfection (GFP mask, Image J). **e.** Immune staining quantification of nuclear P53 (DAPI mask, Image J) in GFP-positive 232 cells (KO cells) transiently transfected as in **d.** and treated for 30 min with 60 μM etoposide and recovered for 6 h, mean intensity ± sem

shown as fold of control-transfected 232P cells, n >100 cells/condition distributed over 5 images. Statistical analysis by independent measures ordinary 2way ANOVA, source of variation between non-transfected and Tau-transfected conditions (in bold), multiple Bonferroni pairwise comparisons among lines for each treatment (in italics), among treatment for each line (**b** and **c**, in vertical).

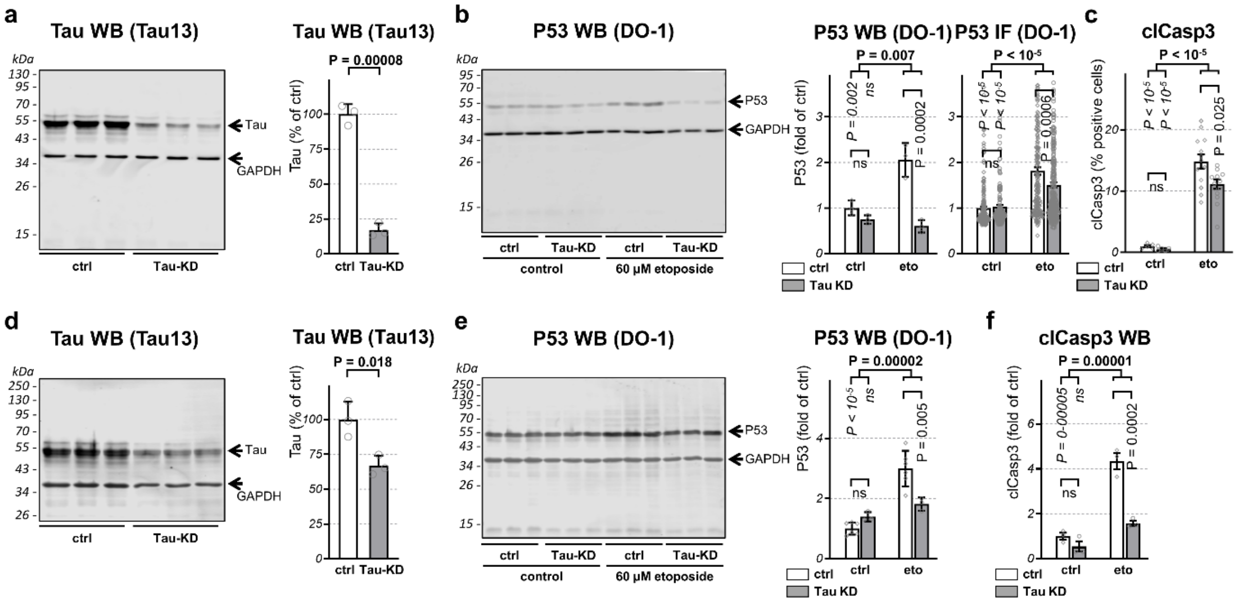

Supplementary Figure 4. Reduced etoposide-induced P53 protein in Tau-KD IMR5 and IMR32 cells. a. Cell lysates obtained from IMR5 cells transduced with the parental shRNA plasmid (ctrl) or with the Tau shRNA plasmid 2112 (Tau-KD) were analyzed by western blot for Tau expression. The Tau signal was normalized for GAPDH and reported as percent of the control, mean +/- SD, n = 3 biological replicates. Unpaired student t-test. **b.** The two IMR5 cell lines at basal conditions or treated 30 min with 15 μM etoposide and recovered for 6 h were analyzed by western blot with the P53 DO-1 antibody. The Tau signal was normalized for GAPDH and reported as fold of the control, mean +/- SD, n = 3 biological replicates. Same conditions were applied to quantify immune stained P53, mean intensity +/- sem of single cell nuclear P53 staining (DAPI mask, ImageJ) shown as fold of untreated control cells (ctrl), n >100 cells/condition distributed over 5 images. **c**. Apoptotic cells were determined by clCasp3 immune staining and reported as percent clCasp3-positive cells of total DAPI-positive cells, mean ± SD of 5 images for the untreated cells (ctrl) and of 15 images for etoposide-treated cells (15 μM eto), n >500 cells/condition. **d**. Same as in **a.** for IMR32 cells transduced with the parental shRNA plasmid (ctrl) or with Tau shRNA plasmid 3127 (Tau-KD). **e.** Same as in **b.** for the western blot analysis. **f**.

Same as in **c.** for 15 and 17 kDa clCasp3 fragments determined by western blot, mean +/- SD (n = 3 biological triplicates). Statistical analysis by independent measures ordinary 2way ANOVA, source of variation for cell lines (in bold), multiple Bonferroni pairwise comparisons for treatment between lines (in italics) or for each line (in vertical).

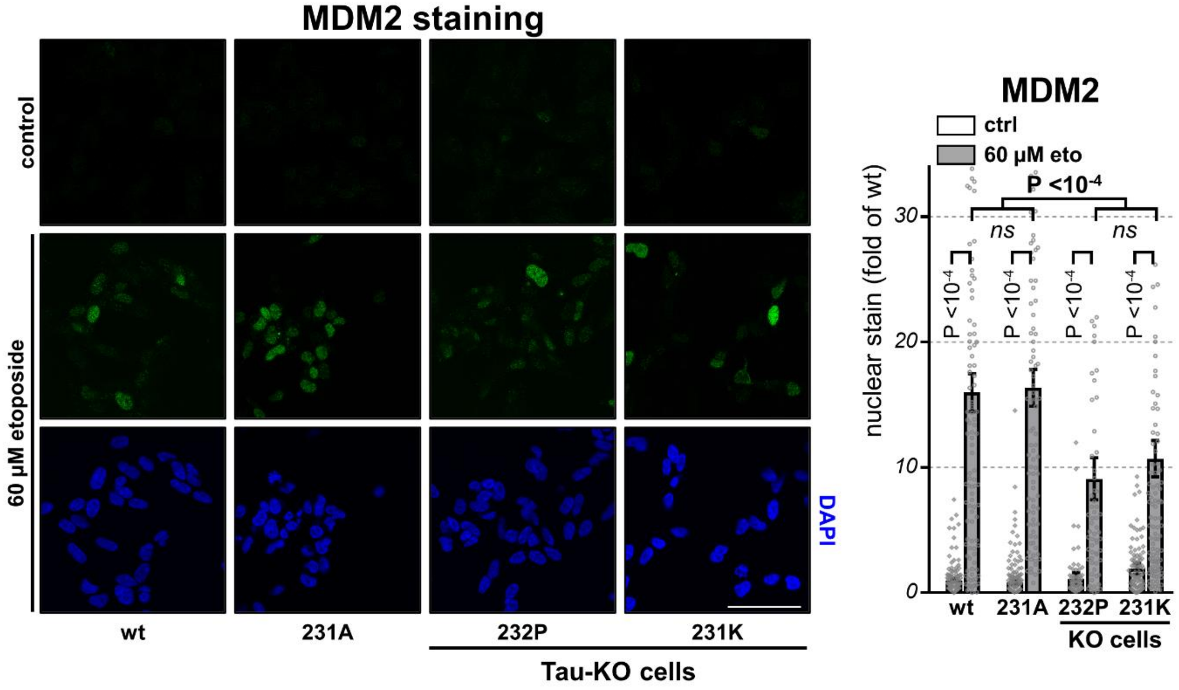

Supplementary Figure 5. Tau deficiency affects MDM2. a. Representative images by laser confocal microscopy of parental (wt) or CRISPR-Cas9 cell lines immune stained with the rabbit MDM2 antibody and counter-stained with DAPI. Cells at basal conditions (control) or after 30 min 60 µM etoposide and 6 h recovery, scale bar 50 µm. Mean intensity +/- sem of single cell nuclear MDM2 staining (DAPI mask, ImageJ) shown as fold of wt cells at basal conditions, n >90 cells/condition distributed over 5 images. Statistical analysis by independent measures ordinary 2way ANOVA, source of variation for genotype (in bold), multiple Bonferroni pairwise comparisons for etoposide treatment between lines with same genotype (in italics) or for the etoposide treatment for each line (in vertical).

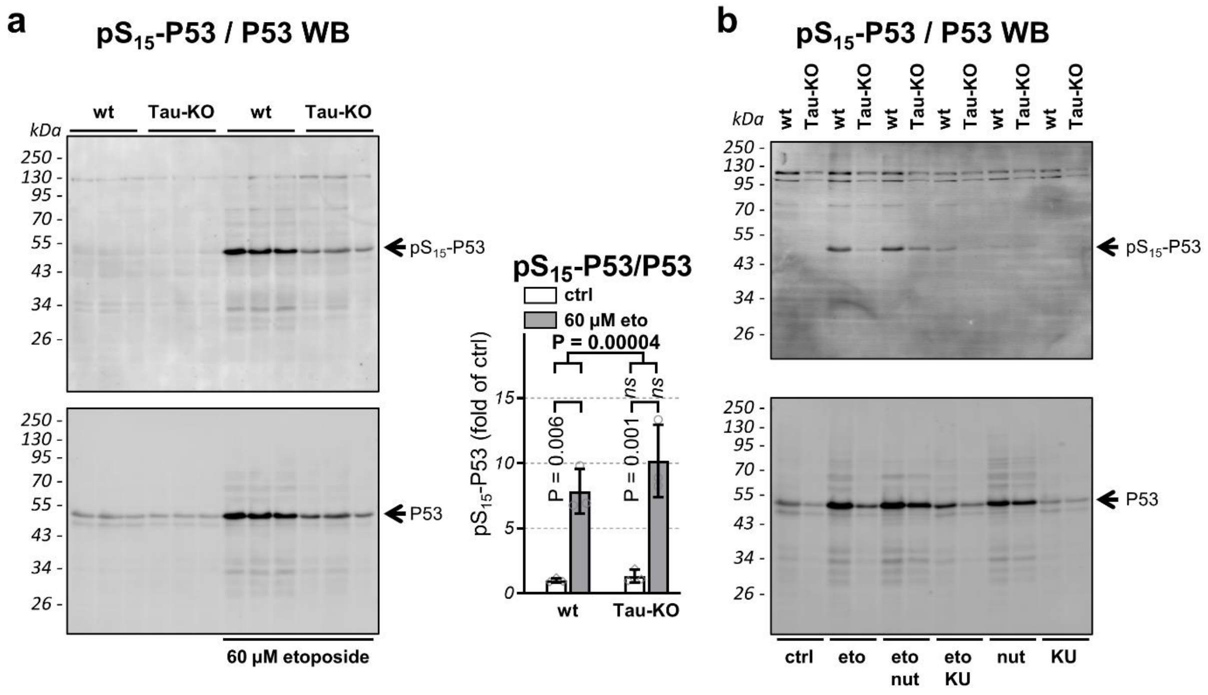

Supplementary Figure 6. Analysis of pS15-P53 phosphorylation. a. Parental (wt) or 232P (Tau-KO) cells at basal conditions or after 30 min 60 μM etoposide and 6 h recovery were analyzed for pS15-P53 or P53 by western blot, n = 3 biological triplicates. Quantification was performed by measuring the signal intensity of pS15-P53 normalized for the signal of total P53, mean +/- SD shown as percent of control (parental cells at basal conditions). Statistical analysis by independent measures ordinary 2way ANOVA, source of variation for cell lines (in bold), multiple Bonferroni pairwise comparisons among lines for each treatment (in italics), or of treatment for each line (in vertical). **b.** Parental (wt) or 232P (Tau-KO) cells at basal conditions (ctrl) or analyzed after 6 h recovery from a 30 min treatment in the absence or presence of 60 μM etoposide in the absence (eto), the recovery was performed in the absence or presence of 5 µg/mL nutlin-3 (eto nut) or 10 µg/mL KU-55933 (eto KU) as indicated. Analysis by western blot for pS15-P53 or P53, a representative experiment out of three experiments is shown.

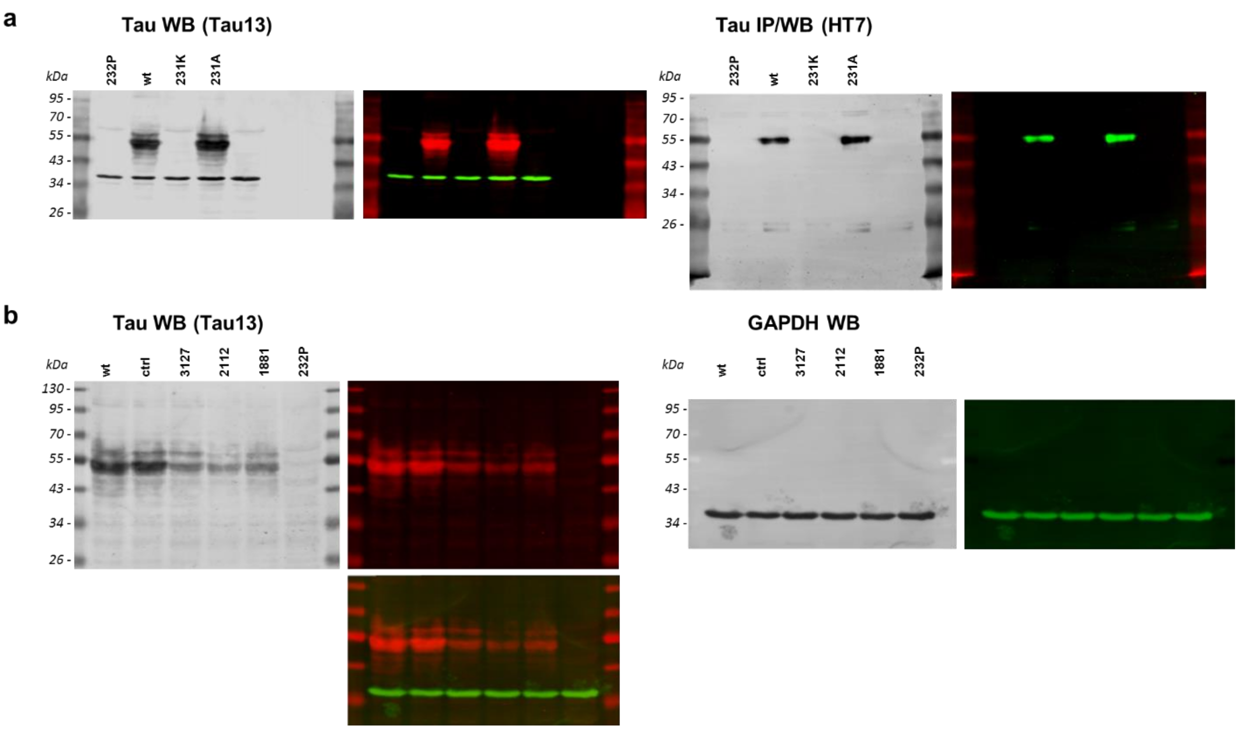

Supplementary Figure 7. Unprocessed western blot images (in grey tones) used for creating the corresponding panels in Fig.1a and 1b. Shown are also the original dual fluorescence images.

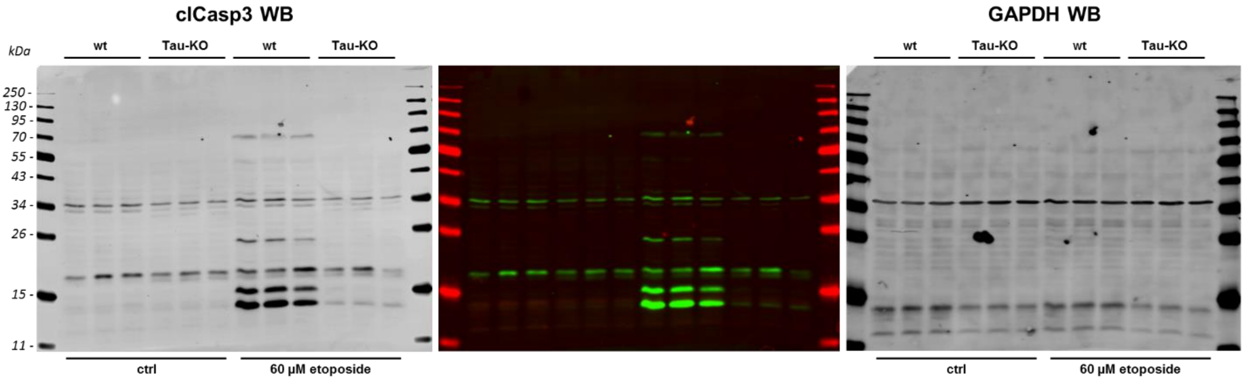

Supplementary Figure 8. Unprocessed western blot images (in grey tones) used for creating the corresponding panels in Fig.2. Shown are also the original dual fluorescence image.

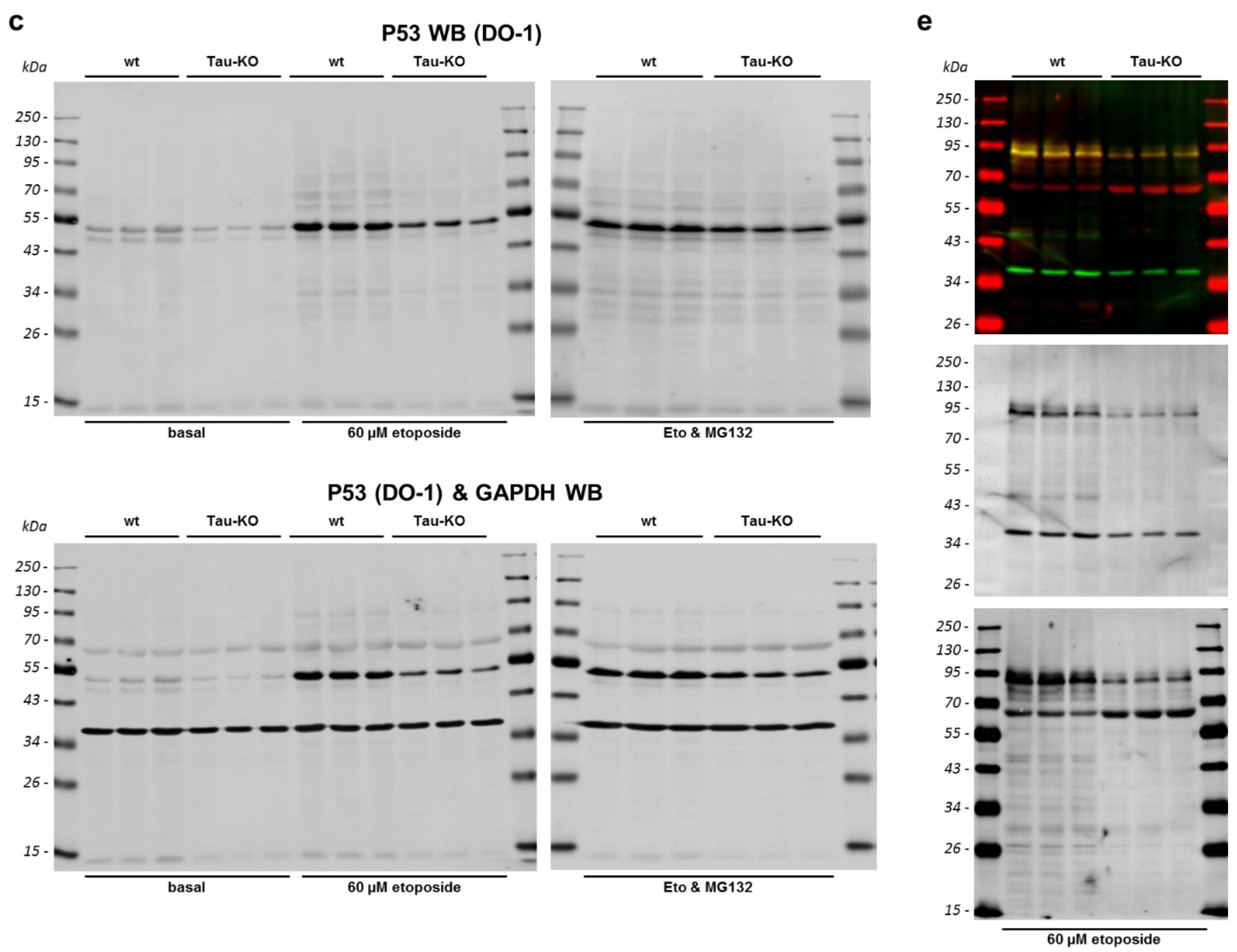

Supplementary Figure 9. Unprocessed western blot images used for creating the corresponding panels in Fig.7c and 7e.

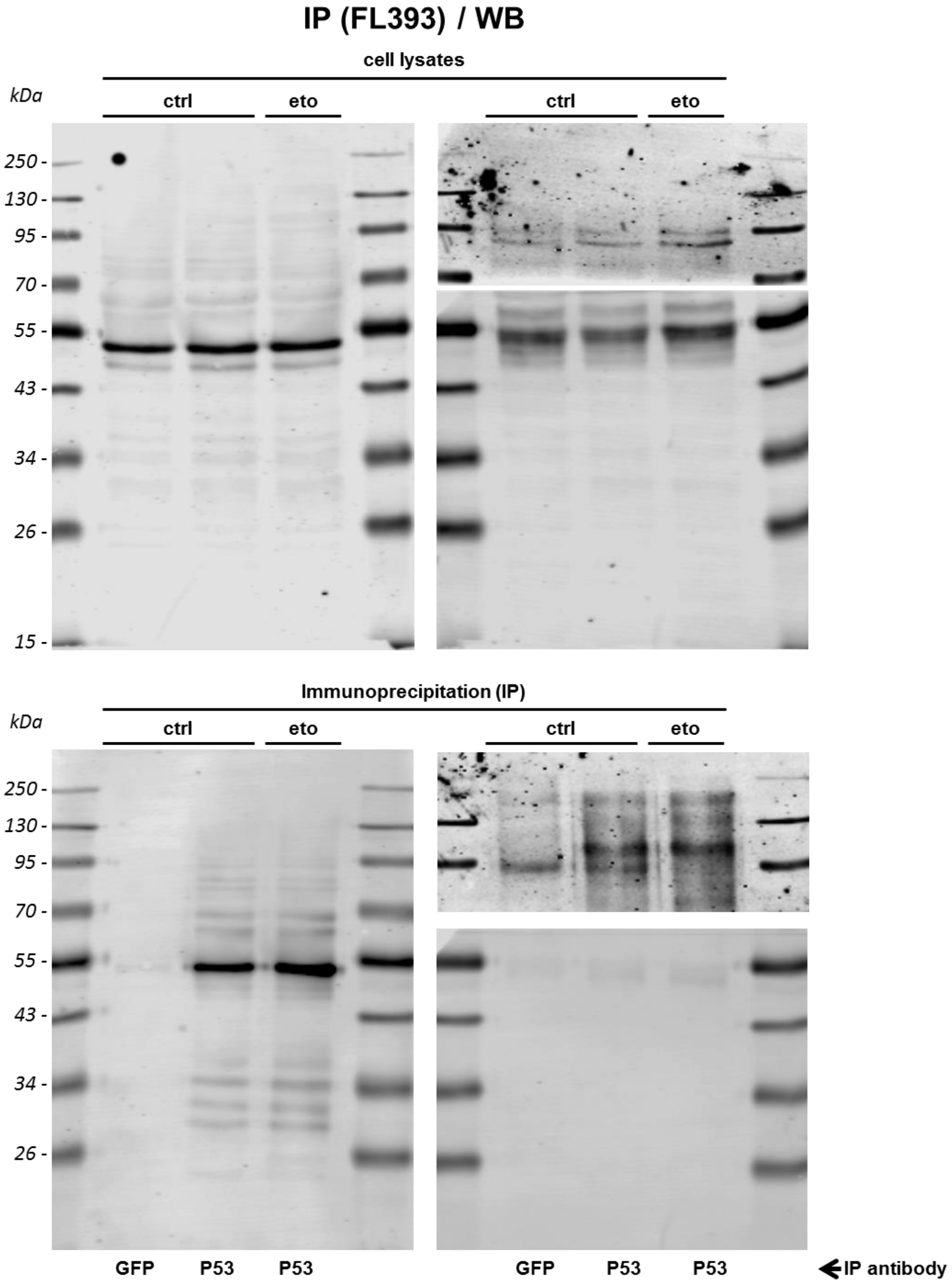

Supplementary Figure 10. Unprocessed western blot images used for creating the corresponding panels in Fig.8. The MDM2 and Tau blots shown on the left were cut between the 55 kDa and the 95 kDa protein size markers and analyzed separately.

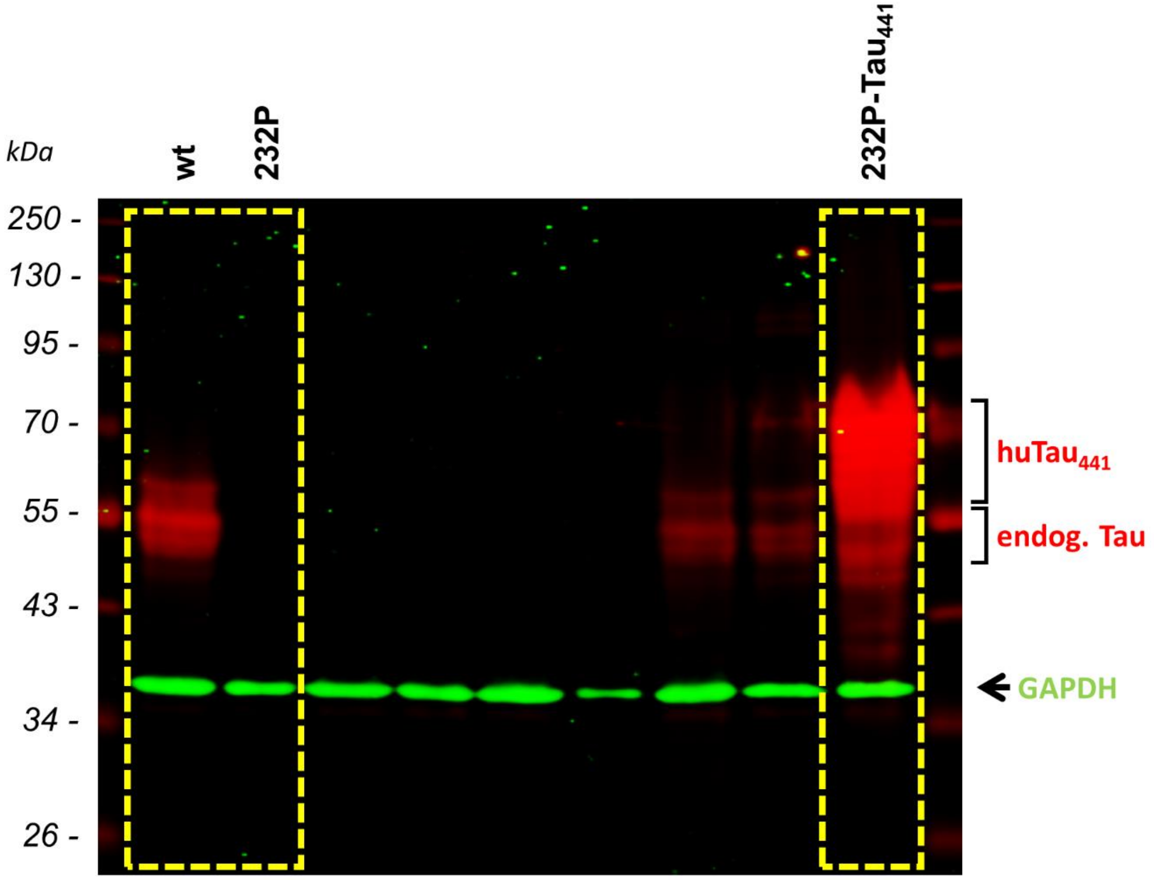

Supplementary Figure 11. Unprocessed gel image used for creating Supplementary Fig.3a.
