# Supplementary material for: Tau affects P53 function and cell fate during the DNA damage response
Source: Commun Biol. 2020 May 19;3:245. doi: 10.1038/s42003-020-0975-4 (PMC7237658; doi:10.1038/s42003-020-0975-4)
Supplement: Supplementary file 3 — Supplementary Data 2 [file 42003_2020_975_MOESM3_ESM.pdf]

DNA SEQUENCING DATA FIGURE 1

Microsynth raw sequencing data:

Clone 231A: →all sequences corresponding to three different colonies show WT form of Tau

231A B WT MAEPRQEFEVMEDHAGTYGLGDRKDQGGYTMHQDQEGDTDAGLK…

231A E WT MAEPRQEFEVMEDHAGTYGLGDRKDQGGYTMHQDQEGDTDAGLK…

231A L WT MAEPRQEFEVMEDHAGTYGLGDRKDQGGYTMHQDQEGDTDAGLK…

>231A B_CMV-for

CTGCTTACTGGCTTATCGAAATTAATACGACTCACTATAGGGAGACCCAAGCTTGGTACCGAGCTCGGATCCGTGAAC
TTTGAACCAGGATGGCTGAGCCCCGCCAGGAGTTCGAAGTGATGGAAGATCACGCTGGGACGTACGGGTTGGGGGA
CAGGAAAGATCAGGGGGGCTACACCATGCACCAAGACCAAGAGGGTGACACGGACGCTGGCCTGAAAGGTTAGTGG
ACAGCCATGCACAGCAGGCCCAGATCACTGCAAGCCAAGGGGTGGCGGGAACAGTTTGCATCCAGAATTGCAAAGA
AATTTTAAATACATTATTGTCTTAGACTGTCAGTAAAGTAAAGCCTCATTAATTTGAGTGGGCCAAGATAACTCAAGCA
GTGAGATAATGGCCAGACACGGTGGCTCACGCCTGTAATCTCGAGCATGCATCTAGAGGGCCCTATTCTATAGTGTCA
CCTAAATGCTAGAGCTCGCTGATCAGCCTCGACTGTGCCTTCTAGTTGCCAGCCATCTGTTGTTTGCCCCTCCCCCGTGC
CTTCCTTGACCCTGGAAGGTGCCACTCCCACTGTCCTTTCCTAATAAAATGAGGAAATTGCATCGCATTGTCTGAGTAG
GTGTCATTCTATTCTGGGGGGTGGGGTGGGGCAGGACAGCAAGGGGGAGGATTGGGAAGACAATAGCAGGCATGC
TGGGGATGCGGTGGGCTCTATGGCTTCTGAGGCGGAAAGAACCAGCTGGGGCTCTAGGGGGTATCCCCACGCGCCC
TGTAGCGGCGCATTAAGCGCGGCGGGTGTGGTGGTTACGCGCAGCGGGACCGSTAMACTTGCCAGCGCCCTAGCGC
CCGCTCCTTTCACTTACTTCCCTTCCTTTCTCGCCACGTTCGCCGGCTTTCCCCGACAAGCT

>231A E_CMV-for

AGAGCTCTCTGGCTAACTAGAGAACCCACTGCTTACTGGCTTATCGAAATTAATACGACTCACTATAGGGAGACCCAA
GCTTGGTACCGAGCTCGGATCCGTGAACTTTGAACCAGGATGGCTGAGCCCCGCCAGGAGTTCGAAGTGATGGAAGA
TCACGCTGGGACGTACGGGTTGGGGGACAGGAAAGATCAGGGGGGCTACACCATGCACCAAGACCAAGAGGGTGA
CACGGACGCTGGCCTGAAAGGTTAGTGGACAGCCATGCACAGCAGGCCCAGATCACTGCAAGCCAAGGGGTGGCGG
GAACAGTTTGCATCCAGAATTGCAAAGAAATTTTAAATACATTATTGTCTTAGACTGTCAGTAAAGTAAAGCCTCATTA
ATTTGAGTGGGCCAAGATAACTCAAGCAGTGAGATAATGGCCAGACACGGTGGCTCACGCCTGTAATCTCGAGCATG
CATCTAGAGGGCCCTATTCTATAGTGTCACCTAAATGCTAGAGCTCGCTGATCAGCCTCGACTGTGCCTTCTAGTTGCC
AGCCATCTGTTGTTTGCCCCTCCCCCGTGCCTTCCTTGACCCTGGAAGGTGCCACTCCCACTGTCCTTTCCTAATAAAAT
GAGGAAATTGCATCGCATTGTCTGAGTAGGTGTCATTCTATTCTGGGGGGTGGGGTGGGGCAGGACAGCAAGGGGG
AGGATTGGGAAGACAATAGCAGGCATGCTGGGGATGCGGTGGGCTCTATGGCTTCTGAGGCGGAAAGAACCAGCTG
GGGCTCTAGGGGGTATCCCCACGCGCCCTGTAGCGGCGCATTAACCGCGGCGGGTGTGGTGGTTACTCGCAGCGTGA
CCGCTACACTTGCCAGCGCCCTAGCGCCCGCTCCTTTCGCTTTCTTCCCTTCCTTTCTCGCCACGTTCGCCGGCTTTCCCG
TCAAGCTCTAAATCGGGGGCTCCCTTTWAGGGTTCCGATTTAGTGCT

>231A L_CMV-for

AGAGAACCCACTGCTTACTGGCTTATCGAAATTAATACGACTCACTATAGGGAGACCCAAGCTTGGTACCGAGCTCGG
ATCCGTGAACTTTGAACCAGGATGGCTGAGCCCCGCCAGGAGTTCGAAGTGATGGAAGATCACGCTGGGACGTACG
GGTTGGGGGACAGGAAAGATCAGGGGGGCTACACCATGCACCAAGACCAAGAGGGTGACACGGACGCTGGCCTGA
AAGGTTAGTGGACAGCCATGCACAGCAGGCCCAGATCACTGCAAGCCAAGGGGTGGCGGGAACAGTTTGCATCCAG
AATTGCAAAGAAATTTTAAATACATTATTGTCTTAGACTGTCAGTAAAGTAAAGCCTCATTAATTTGAGTGGGCCAAGA

TAACTCAAGCAGTGAGATAATGGCCAGACACGGTGGCTCACGCCTGTAATCTCGAGCATGCATCTAGAGGGCCCTATT
CTATAGTGTCACCTAAATGCTAGAGCTCGCTGATCAGCCTCGACTGTGCCTTCTAGTTGCCAGCCATCTGTTGTTTGCCC
CTCCCCCGTGCCTTCCTTGACCCTGGAAGGTGCCACTCCCACTGTCCTTTCCTAATAAAATGAGGAAATTGCATCGCATT
GTCTGAGTAGGTGTCATTCTATTCTGGGGGGTGGGGTGGGGCAGGACAGCAAGGGGGAGGATTGGGAAGACAATA
GCAGGCATGCTGGGGATGCGGTGGGCTCTATGGCTTCTGAGGCGGAAAGAACCAGCTGGGGCTCTAGGGGGTATCC
CCACGCGCCCTGTAGCGGCGCATTAAGCGCGGCGGGTGTGGTGGTTACGCGCAGCGTGACCGCTACACTTGCCAGCG
CCCTAGCGCCCGCTCCTTTCGCTTTCTTCCCTTCCTTTCTCGCCACGTTCGCCGGCTTTCCCCGTCAAGCTCTAAATCGGG
GGCTCCCTTTAGGGTTCCGATTTAGTGCTTTACGGCACCTCGACCCCCAAAAACTTGAATAGGGGTGATGGTTCMCGT
AGTGGGCCATCGCCCTGATAGACGGTTTTTCCCCCTT

Clone 231K: →sequences corresponding to two different colonies

231K A 1 bp insertion MAEPRQEFEVMEDHAGTYGVGGQERSGGLHHAPRPRGstopcodon

231K L 5bp deletion MAEPRQEFEVMEDHAGTYGGQERSGGLHHAPRPRGstopcodon

>231K A_CMV-for

CTCTCTGGCTAACTAGAGAACCCACTGCTTACTGGCTTATCGAAATTAATACGACTCACTATAGGGAGACCCAAGCTTG
GTACCGAGCTCGGATCCGTGAACTTTGAACCAGGATGGCTGAGCCCCGCCAGGAGTTCGAAGTGATGGAAGATCACG
CTGGGACGTACGGGGTTGGGGGACAGGAAAGATCAGGGGGGCTACACCATGCACCAAGACCAAGAGGGTGACACG
GACGCTGGCCTGAAAGGTTAGTGGACAGCCATGCACAGCAGGCCCAGATCACTGCAAGCCAAGGGGTGGCGGGAAC
AGTTTGCATCCAGAATTGCAAAGAAATTTTAAATACATTATTGTCTTAGACTGTCAGTAAAGTAAAGCCTCATTAATTT
GAGTGGGCCAAGATAACTCAAGCAGTGAGATAATGGCCAGACACGGTGGCTCACGCCTGTAATCTCGAGCATGCATC
TAGAGGGCCCTATTCTATAGTGTCACCTAAATGCTAGAGCTCGCTGATCAGCCTCGACTGTGCCTTCTAGTTGCCAGCC
ATCTGTTGTTTGCCCCTCCCCCGTGCCTTCCTTGACCCTGGAAGGTGCCACTCCCACTGTCCTTTCCTAATAAAATGAGG
AAATTGCATCGCATTGTCTGAGTAGGTGTCATTCTATTCTGGGGGGTGGGGTGGGGCAGGACAGCAAGGGGGAGGA
TTGGGAAGACAATAGCAGGCATGCTGGGGATGCGGTGGGCTCTATGGCTTCTGAGGCGGAAAGAACCAGCTGGGGC
TCTAGGGGGTATCCCCACGCGCCCTGTAGCGGCGCATTAAGCGCGGCGGGTGTGGTGGTTACGCGCAGCGTGACCGC
TACACTTGCCAGCGCCCTAGCGCCCGCTCCTTTCGCTTTCTTCCCTTCCTTTCTCGCCACGTTCGCCGGCTTTCCCCGTCA
AGCTCTAAATCGGGGGCTCCCTTTAGGGTTCCGATTTAGTGCTTTACGGCACCTCGACCCCAAAAAACTTGATTAGGGT
GATGGTTCACGTAGTGGGCCATCGCCCTGATAGACGGTTTTTCGCCCTTTGACGTTGGAGTCCACGTTCTTTAAWART
GGACTCTTGTTCCAAACTGGAACAAMMCTCAACCCTATCYCGGTCTATTCTTTTGATTTWWAAGGGATTTTGCCNATT
TCGGCCTATTGGTTAAAAATNRRCTGATTTA

>231K L_CMV-for

GAGCTCTCTGGCTAACTAGAGAACCCACTGCTTACTGGCTTATCGAAATTAATACGACTCACTATAGGGAGACCCAAG
CTTGGTACCGAGCTCGGATCCGTGAACTTTGAACCAGGATGGCTGAGCCCCGCCAGGAGTTCGAAGTGATGGAAGAT
CACGCTGGGACGTACGGGGGACAGGAAAGATCAGGGGGGCTACACCATGCACCAAGACCAAGAGGGTGACACGGA
CGCTGGCCTGAAAGGTTAGTGGACAGCCATGCACAGCAGGCCCAGATCACTGCAAGCCAAGGGGTGGCGGGAACAG
TTTGCATCCAGAATTGCAAAGAAATTTTAAATACATTATTGTCTTAGACTGTCAGTAAAGTAAAGCCTCATTAATTTGA
GTGGGCCAAGATAACTCAAGCAGTGAGATAATGGCCAGACACGGTGGCTCACGCCTGTAATCTCGAGCATGCATCTA
GAGGGCCCTATTCTATAGTGTCACCTAAATGCTAGAGCTCGCTGATCAGCCTCGACTGTGCCTTCTAGTTGCCAGCCAT
CTGTTGTTTGCCCCTCCCCCGTGCCTTCCTTGACCCTGGAAGGTGCCACTCCCACTGTCCTTTCCTAATAAAATGAGGAA
ATTGCATCGCATTGTCTGAGTAGGTGTCATTCTATTCTGGGGGGTGGGGTGGGGCAGGACAGCAAGGGGGAGGATT
GGGAAGACAATAGCAGGCATGCTGGGGATGCGGTGGGCTCTATGGCTTCTGAGGCGGAAAGAACCAGCTGGGGCTC
TAGGGGGTATCCCCACGCGCCCTGTAGCGGCGCATTAAGCGCGGCGGGTGTGGTGGTTACGCGCAGCGTGACCGCT
ACACTTGCCAGCGCCCTAGCGCCCGCTCCTTTCGCTTTCTTCCCTTCCTTTCTCGCCACGTTCGCCGGCTTTCCCCGTCAA
GCTCTAAATCGGGGGCTCCCTTTAGGGTTCCGATTTAGTGCTTTACGGCACCTCGACCCCAAAAAACTTGATTAGGGT
GATGGTTCACGTAGTGGGCCATCGCCCTGATAGACGGTTTTTCGCCCTTTGACGTTGGAGTCCACGTTCTTTAATAGTG
GACTCTTGTTCCAAACTGGAACAACMCTCAACCCTATCTCGGTCTATTCTTTTGATTATAAGGGATTTTGCCG

Clone 232P: → sequences corresponding to four different colonies

232P E big insertion MAEPRQEFEVQQKLFLNLPSLLLSLstopcodon

232P G 1bp insertion MAEPRQEFEADGRSRWDVRVGGQERSGGLHHAPRPRGstopcodon

232P M big insertion MAEPRQEFEVQQKLFLNLPSLLLSLstopcodon

232P O 1bp insertion MAEPRQEFEADGRSRWDVRVGGQERSGGLHHAPRPRGstopcodon-

>232P-E_CMV-for

GCTCTCTGGCTAACTAGAGAACCCACTGCTTACTGGCTTATCGAAATTAATACGACTCACTATAGGGAGACCCAAGCTT
GGTACCGAGCTCGGATCCGTGAACTTTGAACCAGGATGGCTGAGCCCCGCCAGGAGTTCGAAGTACAGCAAAAACTA
TTCTTAAACCTACCAAGCCTCCTACTATCATTATGAATAATTTTATATACCACAGCCAATTTGTTATGTTAAACCAATTCC
ACAAACTTGCCCATTTATCTAATTCCAATAATTCTTGTTCATTCTTTTCTTGCTGGTTTTGCGATTCTTCAATTAAGGAGT
GTATTAAGCTTGTGTAATTGTTAATTTCTCTGTCCCACTCCATCCAGGTCGTGTGATTCCGATGCTATTGCTTTATTTGTA
ACCATTATAAGCTGCAATAAACAAGTTGGGGTGGGCGAAGAACTCCAGCATGAGATCCCCGCGCTGGAGGATCATCC
AGCCGTGGGGGCGGGAGTTCGCCCTGCGCGACCCGGCCGGCAACTGCGTGCACTTCGTGGCCGAGGAGCAGGACTG
ACACGTGCTACGAGATTTCGATTCCACCGCCGCCTTCTATGAAAGGTTGGGCTTCGGAATCGTTTTCCGGGACGCCGG
CTGGACGTGGTGTAATTCTTTATATTATGATGGAAGATCACGCTGGGACGTACGGGTTGGGGGACAGGAAAGATCAG
GGGGGCTACACCATGCACCAAGACCAAGAGGGTGACACGGACGCTGGCCTGAAAGGTTAGTGGACAGCCATGCACA
GCAGGCCCAGATCACTGCAAGCCAAGGGGTGGCGGGAACAGTTTGCATCCAGAATTGCAAAGAAATTTTAAATACAT
TATTGTCTTAGACTGTCAGTAAAGTAAAGCCTCATTAATTTGAGTGGGCCAAGATAACTCAAGCAGTGAGATAATGGC
CAGACACGGTGGCTCACGCCTGTAATCTCGAGCATGCATCTAGAGGGCCCTATTCTATAGTGTCACCTAAATGCTAGA
GCTCGCTGATCAGCCTCGACTGTGCCTTCTAGTTGCCAGCCATCTGTTGTTTGCCCCTCCCCCGTGCCTTCCTTGACCCT
GGAAGGTGCCMCTCCCACTGTCCTTTCCTAATAAAATGAGGAAATTGCATCGCATNGTCTG

>232P-G_CMV-for

GCTCTCTGGCTAACTAGAGAACCCACTGCTTACTGGCTTATCGAAATTAATACGACTCACTATAGGGAGACCCAAGCTT
GGTACCGAGCTCGGATCCGTGAACTTTGAACCAGGATGGCTGAGCCCCGCCAGGAGTTCGAAGCTGATGGAAGATCA
CGCTGGGACGTACGGGTTGGGGGACAGGAAAGATCAGGGGGGCTACACCATGCACCAAGACCAAGAGGGTGACAC
GGACGCTGGCCTGAAAGGTTAGTGGACAGCCATGCACAGCAGGCCCAGATCACTGCAAGCCAAGGGGTGGCGGGAA
CAGTTTGCATCCAGAATTGCAAAGAAATTTTAAATACATTATTGTCTTAGACTGTCAGTAAAGTAAAGCCTCATTAATTT
GAGTGGGCCAAGATAACTCAAGCAGTGAGATAATGGCCAGACACGGTGGCTCACGCCTGTAATCTCGAGCATGCATC
TAGAGGGCCCTATTCTATAGTGTCACCTAAATGCTAGAGCTCGCTGATCAGCCTCGACTGTGCCTTCTAGTTGCCAGCC
ATCTGTTGTTTGCCCCTCCCCCGTGCCTTCCTTGACCCTGGAAGGTGCCACTCCCACTGTCCTTTCCTAATAAAATGAGG
AAATTGCATCGCATTGTCTGAGTAGGTGTCATTCTATTCTGGGGGGTGGGGTGGGGCAGGACAGCAAGGGGGAGGA
TTGGGAAGACAATAGCAGGCATGCTGGGGATGCGGTGGGCTCTATGGCTTCTGAGGCGGAAAGAACCAGCTGGGGC
TCTAGGGGGTATCCCCACGCGCCCTGTAGCGGCGCATTAAGCGCGGCGGGTGTGGTGGTTACGCGCAGCGTGACCGC
TACACTTGCCAGCGCCCTAGCGCCCGCTCCTTTCGCTTTCTTCCCTTCCTTTCTCGCCACGTTCGCCGGCTTTCCCCGTCAA
GCTCTAAATCGGGGGCTCCCTTTAGGGTTCCGATTTAGTGCTTTACGGCACCTCGACCCCAAAAAACTTGATTAGGGTG
ATGGTTCACGTAGTGGGCCATCGCCCTGATAGACGGTTTTTCGCCCTTTGACGTTGGAGTCCACGTTCTTTAATAGTGG
ACTCTTGTTCCAAACTGGAACAACMCTCAACCCTWTYTCGGTCTATTCTTTTGATTTWTAAGGGAATTTGCCGATTTCG
NCCTNTGGTTAAAAATGAGCTGATTTAAMAAAATTTA

>232P-M_CMV-for

AGCTCTCTGGCTAACTAGAGAACCCACTGCTTACTGGCTTATCGAAATTAATACGACTCACTATAGGGAGACCCAAGCT
TGGTACCGAGCTCGGATCCGTGAACTTTGAACCAGGATGGCTGAGCCCCGCCAGGAGTTCGAAGTACAGCAAAAACT
ATTCTTAAACCTACCAAGCCTCCTACTATCATTATGAATAATTTTATATACCACAGCCAATTTGTTATGTTAAACCAATTC
CACAAACTTGCCCATTTATCTAATTCCAATAATTCTTGTTCATTCTTTTCTTGCTGGTTTTGCGATTCTTCAATTAAGGAG
TGTATTAAGCTTGTGTAATTGTTAATTTCTCTGTCCCACTCCATCCAGGTCGTGTGATTCCGATGCTATTGCTTTATTTGT
AACCATTATAAGCTGCAATAAACAAGTTGGGGTGGGCGAAGAACTCCAGCATGAGATCCCCGCGCTGGAGGATCATC
CAGCCGTGGGGGCGGGAGTTCGCCCTGCGCGACCCGGCCGGCAACTGCGTGCACTTCGTGGCCGAGGAGCAGGACT
GACACGTGCTACGAGATTTCGATTCCACCGCCGCCTTCTATGAAAGGTTGGGCTTCGGAATCGTTTTCCGGGACGCCG
GCTGGACGTGGTGTAATTCTTTATATTATGATGGAAGATCACGCTGGGACGTACGGGTTGGGGGACAGGAAAGATCA
GGGGGGCTACACCATGCACCAAGACCAAGAGGGTGACACGGACGCTGGCCTGAAAGGTTAGTGGACAGCCATGCAC
AGCAGGCCCAGATCACTGCAAGCCAAGGGGTGGCGGGAACAGTTTGCATCCAGAATTGCAAAGAAATTTTAAATACA
TTATTGTCTTAGACTGTCAGTAAAGTAAAGCCTCATTAATTTGAGTGGGCCAAGATAACTCAAGCAGTGAGATAATGG
CCAGACACGGTGGCTCACGCCTGTAATCTCGAGCATGCATCTAGAGGGCCCTATTCTATAGTGTCACCTAAATGCTAG
AGCTCGCTGATCAGCCTCGACTGTGCCTTCTAGTTGCCAGCCATCTGTTGTTTGCCCCTCCCCCGTGCCTTCCTTGACCC
TGGAAGGKGCCACTCCCACTGTCCTTTCCTAATAAAATGAGGAAATTGCATCGCATTGTCTGAGTAGGKGTCATTCNAT
TCTGGGGGGKKGGGTTGGGGCAGGAMNGCCAAGGGGAAGA

>232P-O_CMV-for

AGAGCTCTCTGGCTACTAGAGAACCCACTGCTTACTGGCTTATCGAAATTAATACGACTCACTATAGGGAGACCCAAG
CTTGGTACCGAGCTCGGATCCGTGAACTTTGAACCAGGATGGCTGAGCCCCGCCAGGAGTTCGAAGCTGATGGAAGA
TCACGCTGGGACGTACGGGTTGGGGGACAGGAAAGATCAGGGGGGCTACACCATGCACCAAGACCAAGAGGGTGA
CACGGACGCTGGCCTGAAAGGTTAGTGGACAGCCATGCACAGCAGGCCCAGATCACTGCAAGCCAAGGGGTGGCGG
GAACAGTTTGCATCCAGAATTGCAAAGAAATTTTAAATACATTATTGTCTTAGACTGTCAGTAAAGTAAAGCCTCATTA
ATTTGAGTGGGCCAAGATAACTCAAGCAGTGAGATAATGGCCAGACACGGTGGCTCACGCCTGTAATCTCGAGCATG
CATCTAGAGGGCCCTATTCTATAGTGTCACCTAAATGCTAGAGCTCGCTGATCAGCCTCGACTGTGCCTTCTAGTTGCC
AGCCATCTGTTGTTTGCCCCTCCCCCGTGCCTTCCTTGACCCTGGAAGGTGCCACTCCCACTGTCCTTTCCTAATAAAAT
GAGGAAATTGCATCGCATTGTCTGAGTAGGTGTCATTCTATTCTGGGGGGTGGGGTGGGGCAGGACAGCAAGGGGG
AGGATTGGGAAGACAATAGCAGGCATGCTGGGGATGCGGTGGGCTCTATGGCTTCTGAGGCGGAAAGAACCAGCTG
GGGCTCTAGGGGGTATCCCCACGCGCCCTGTAGCGGCGCATTAAGCGCGGCGGGTGTGGTGGTTACGCGCAGCGTG
ACCGCTACACTTGCCAGCGCCCTAGCGCCCGCTCCTTTCGCTTTCTTCCCTTCCTTTCTCGCCACGTTCGCCGGCTTTCCC
CGTCAAGCTCTAAATCGGGGGCTCCCTTTAGGGTTCCGATTTAGTGCTTTACGGCACCTCGACCCCAAAAAACTTGATT
AGGGTGATGGTTCACGTAGTGGGCCATCGCCCTGATAGACGGTTTTTCGCCCTTTGACGTTGGAGTCCACGTTCTTTA
ATAGTGGACTCTTGTTCCAAACTGGAACAACMCTCAACCCTATCTCGGTCTATTCTTTTGATTTWAAGGGATTTTGCCG
ATTTCGGCNNATTGGTTAAAAAT

PLASMIDS MAPS AND SEQUENCING

shRNA SEQUENCE

pSME-352 shRNA Tau1881:

Full map:

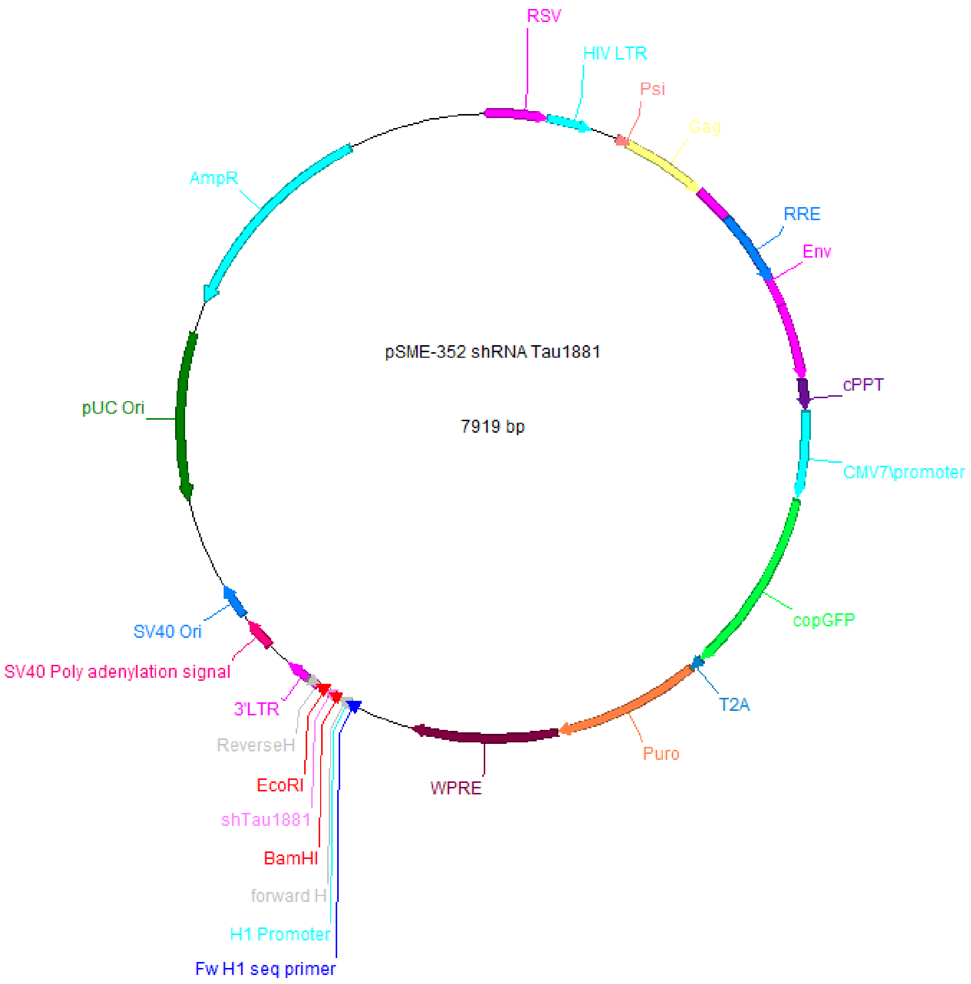

FASTA Sequence information:

>shRNA 1881 A1_H1 Promoter FWD

GATTTGGGATCTTATAAGTTCTGTATGAGACCACTTGGATCCTGGTGAACCTCCAAAATCACTTCCTGTCAGATGATTT
TGGAGGTTCACCATTTTTGAATTCTTCGATTCTGCTTTTTGCTTCTACTGGGTCTCTCTGGTTAGACCAGATCTGAGCCT
GGGAGCTCTCTGGCTAACTAGGGAACCCACTGCTTAAGCCTCAATAAAGCTTGCCTTGAGTGCTTCAAGTAGTGTGTG
CCCGTCTGTTGTGTGACTCTGGTAACTAGAGATCCCTCAGACCCTTTCAGTCAGTGTGGAAAATCTCTAGCAGTAGTAG
TTCATGTCATCTTATTATTCAGTATTTATAACTTGCAAAGAAATGAATATCAGAGAGTGAGAGGAACTTGTTTATTGCA
GCTTATAATGGTTACAAATAAAGCAATAGCATCACAAATTTCACAAATAAAGCATTTTTTTCACTGCATTCTAGTTGTGG
TTTGTCCAAACTCATCAATGTATCTTATCATGTCTGGCTCTAGCTATCCCGCCCCTAACTCCGCCCATCCCGCCCCTAACT
CCGCCCAGTTCCGCCCATTCTCCGCCCCATGGCTGACTAATTTTTTTTATTTATGCAGAGGCCGAGGCCGCCTCGGCCTC
TGAGCTATTCCAGAAGTAGTGAGGAGGCTTTTTTGGAGGCCTAGACTTTTGCAGAGACCAAATTCGTAATCATGTCAT

AGCTGTTTCCTGTGTGAAATTGTTATCCGCTCACAATTCCACACAACATACGAGCCGGAAGCATAAAGTGTAAAGCCT
GGGGTGCCTAATGAGTGAGCTAACTCACATTAATTGCGTTGCGCTCACTGCCCGCTTTCCAGTCGGGAAACCTGTCGT
GCCAGCTGCATTAATGAATCGGCCAACGCGCGGGGAGAGGCGGTTTGCGTATTGGGCGCTCTTCCGCTTCCTCGCTCA
CTGACTCGCTGCGCTCGGTCGTTCGGCTGCGGCGAGCGGTATCAGCTCACTCAAAGGCGGTAATACGGTTATCCACAG
AATCAGGGGATAACGCAGGAAAGAACATGTGAGCAAAAGGCCAGCAAAAGGCCAGGAACCGTAAAAAGGCCGCGT
TGCTGGCGTTTTTCCATAGGCTCCGCCCCCCTGACGAGCATCMCAAAAATCGACSCTCAAGTCAAAGGTGNGAAACCC
GAMMGGACTWWAAGATACCAGGCGTTTCCCCTGGAAGCTCCCYCGKGSSCTYCCTGTTCCRACCTGCCSCTTACGGA
AAC

>shRNA 1881 B1_H1 Promoter FWD

GGATTTGGGATCTTATAAGTTCTGTATGAGACCACTTGGATCCTGGTGAACCTCCAAAATCACTTCCTGTCAGATGATT
TTGGAGGTTCACCATTTTTGAATTCTTCGATTCTGCTTTTTGCTTCTACTGGGTCTCTCTGGTTAGACCAGATCTGAGCC
TGGGAGCTCTCTGGCTAACTAGGGAACCCACTGCTTAAGCCTCAATAAAGCTTGCCTTGAGTGCTTCAAGTAGTGTGT
GCCCGTCTGTTGTGTGACTCTGGTAACTAGAGATCCCTCAGACCCTTTCAGTCAGTGTGGAAAATCTCTAGCAGTAGTA
GTTCATGTCATCTTATTATTCAGTATTTATAACTTGCAAAGAAATGAATATCAGAGAGTGAGAGGAACTTGTTTATTGC
AGCTTATAATGGTTACAAATAAAGCAATAGCATCACAAATTTCACAAATAAAGCATTTTTTTCACTGCATTCTAGTTGTG
GTTTGTCCAAACTCATCAATGTATCTTATCATGTCTGGCTCTAGCTATCCCGCCCCTAACTCCGCCCATCCCGCCCCTAA
CTCCGCCCAGTTCCGCCCATTCTCCGCCCCATGGCTGACTAATTTTTTTTATTTATGCAGAGGCCGAGGCCGCCTCGGCC
TCTGAGCTATTCCAGAAGTAGTGAGGAGGCTTTTTTGGAGGCCTAGACTTTTGCAGAGACCAAATTCGTAATCATGTC
ATAGCTGTTTCCTGTGTGAAATTGTTATCCGCTCACAATTCCACACAACATACGAGCCGGAAGCATAAAGTGTAAAGCC
TGGGGTGCCTAATGAGTGAGCTAACTCACATTAATTGCGTTGCGCTCACTGCCCGCTTTCCAGTCGGGAAACCTGTCG
TGCCAGCTGCATTAATGAATCGGCCAACGCGCGGGGAGAGGCGGTTTGCGTATTGGGCGCTCTTCCGCTTCCTCGCTC
ACTGACTCGCTGCGCTCGGTCGTTCGGCTGCGGCGAGCGGTATCAGCTCACTCAAAGGCGGTAATACGGTTATCCACA
GAATCAGGGGATAACGCAGGAAAGACATGTGAGCAAAAGGCCAGCAAAAGGCCAGGAACCGTAAAAAGGCCSCGTT
GCTGGCGTTTTTCCATAGGCTCCGCCCCCCTGAC

pSME-353 shRNA Tau2112

Full map:

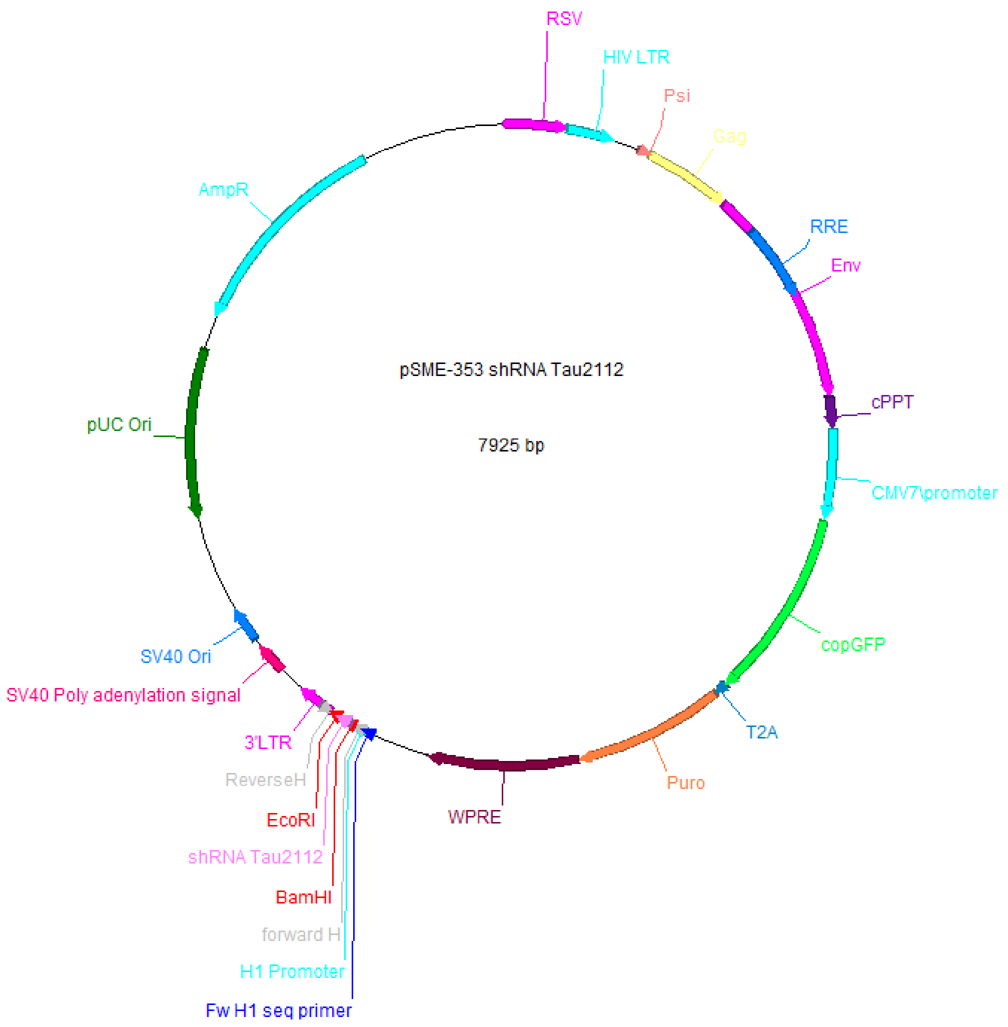

FASTA Sequence information:

>shRNA 2112 A1_H1 Promoter FWD

GATTTGGGATCTTATAAGTTCTGTATGAGACCACTTGGATCCAACTGAGAACCTGAAGCACCAGCTTCCTGTCAGACT
GGTGCTTCAGGTTCTCAGTGTTTTTGAATTCTTCGATTCTGCTTTTTGCTTCTACTGGGTCTCTCTGGTTAGACCAGATCT
GAGCCTGGGAGCTCTCTGGCTAACTAGGGAACCCACTGCTTAAGCCTCAATAAAGCTTGCCTTGAGTGCTTCAAGTAG
TGTGTGCCCGTCTGTTGTGTGACTCTGGTAACTAGAGATCCCTCAGACCCTTTCAGTCAGTGTGGAAAATCTCTAGCAG
TAGTAGTTCATGTCATCTTATTATTCAGTATTTATAACTTGCAAAGAAATGAATATCAGAGAGTGAGAGGAACTTGTTT
ATTGCAGCTTATAATGGTTACAAATAAAGCAATAGCATCACAAATTTCACAAATAAAGCATTTTTTTCACTGCATTCTAG
TTGTGGTTTGTCCAAACTCATCAATGTATCTTATCATGTCTGGCTCTAGCTATCCCGCCCCTAACTCCGCCCATCCCGCC
CCTAACTCCGCCCAGTTCCGCCCATTCTCCGCCCCATGGCTGACTAATTTTTTTTATTTATGCAGAGGCCGAGGCCGCCT
CGGCCTCTGAGCTATTCCAGAAGTAGTGAGGAGGCTTTTTTGGAGGCCTAGACTTTTGCAGAGACCAAATTCGTAATC
ATGTCATAGCTGTTTCCTGTGTGAAATTGTTATCCGCTCACAATTCCACACAACATACGAGCCGGAAGCATAAAGTGTA

AAGCCTGGGGTGCCTAATGAGTGAGCTAACTCACATTAATTGCGTTGCGCTCACTGCCCGCTTTCCAGTCGGGAAACC
TGTCGTGCCAGCTGCATTAATGAATCGGCCAACGCGCGGGGAGAGGCGGTTTGCGTATTGGGCGCTCTTCCGCTTCCT
CGCTCACTGACTCGCTGCGCTCGGTCGTTCGGCTGCGGCGAGCGGTATCAGCTCACTCAAAGGCGGTAATACGGTTAT
CCACAGAATCAGGGGATAACGCAGGAAAGAACATGTGAGCAAAAGGCCAGCAAAAGGCCAGGAACCGTAAAAAGG
CCSCGTTGCTGGCGTTTTTCCATAGGCTCCGCCCCCCTGACGAGCATCMMAAAANTCGACNCT

>shRNA 2112 B1_H1 Promoter FWD

GGATTTGGGATCTTATAAGTTCTGTATGAGACCACTTGGATCCAACTGAGAACCTGAAGCACCAGCTTCCTGTCAGAC
TGGTGCTTCAGGTTCTCAGTGTTTTTGAATTCTTCGATTCTGCTTTTTGCTTCTACTGGGTCTCTCTGGTTAGACCAGATC
TGAGCCTGGGAGCTCTCTGGCTAACTAGGGAACCCACTGCTTAAGCCTCAATAAAGCTTGCCTTGAGTGCTTCAAGTA
GTGTGTGCCCGTCTGTTGTGTGACTCTGGTAACTAGAGATCCCTCAGACCCTTTCAGTCAGTGTGGAAAATCTCTAGCA
GTAGTAGTTCATGTCATCTTATTATTCAGTATTTATAACTTGCAAAGAAATGAATATCAGAGAGTGAGAGGAACTTGTT
TATTGCAGCTTATAATGGTTACAAATAAAGCAATAGCATCACAAATTTCACAAATAAAGCATTTTTTTCACTGCATTCTA
GTTGTGGTTTGTCCAAACTCATCAATGTATCTTATCATGTCTGGCTCTAGCTATCCCGCCCCTAACTCCGCCCATCCCGC
CCCTAACTCCGCCCAGTTCCGCCCATTCTCCGCCCCATGGCTGACTAATTTTTTTTATTTATGCAGAGGCCGAGGCCGCC
TCGGCCTCTGAGCTATTCCAGAAGTAGTGAGGAGGCTTTTTTGGAGGCCTAGACTTTTGCAGAGACCAAATTCGTAAT
CATGTCATAGCTGTTTCCTGTGTGAAATTGTTATCCGCTCACAATTCCACACAACATACGAGCCGGAAGCATAAAGTGT
AAAGCCTGGGGTGCCTAATGAGTGAGCTAACTCACATTAATTGCGTTGCGCTCACTGCCCGCTTTCCAGTCGGGAAAC
CTGTCGTGCCAGCTGCATTAATGAATCGGCCAACGCGCGGGGAGAGGCGGTTTGCGTATTGGGCGCTCTTCCGCTTCC
TCGCTCACTGACTCGCTGCGCTCGGTCGTTCGGCTGCGGCGAGCGGTATCAGCTCACTCAAAGGCGGTAATACGGTTA
TCCACAGAATCAGGGGATAACGCAGGAAAGAACATGTGAGCAAAAGGCCAGCAAAAGGCCAGGAACCGTAAAAAG
GCCGCGTTGCTGGCGTTTTTCCTAGGCTCCGCCCCCCTGACGAGCATCMCAAAAATCGA

pSME-354 shRNA Tau3127:

Full map:

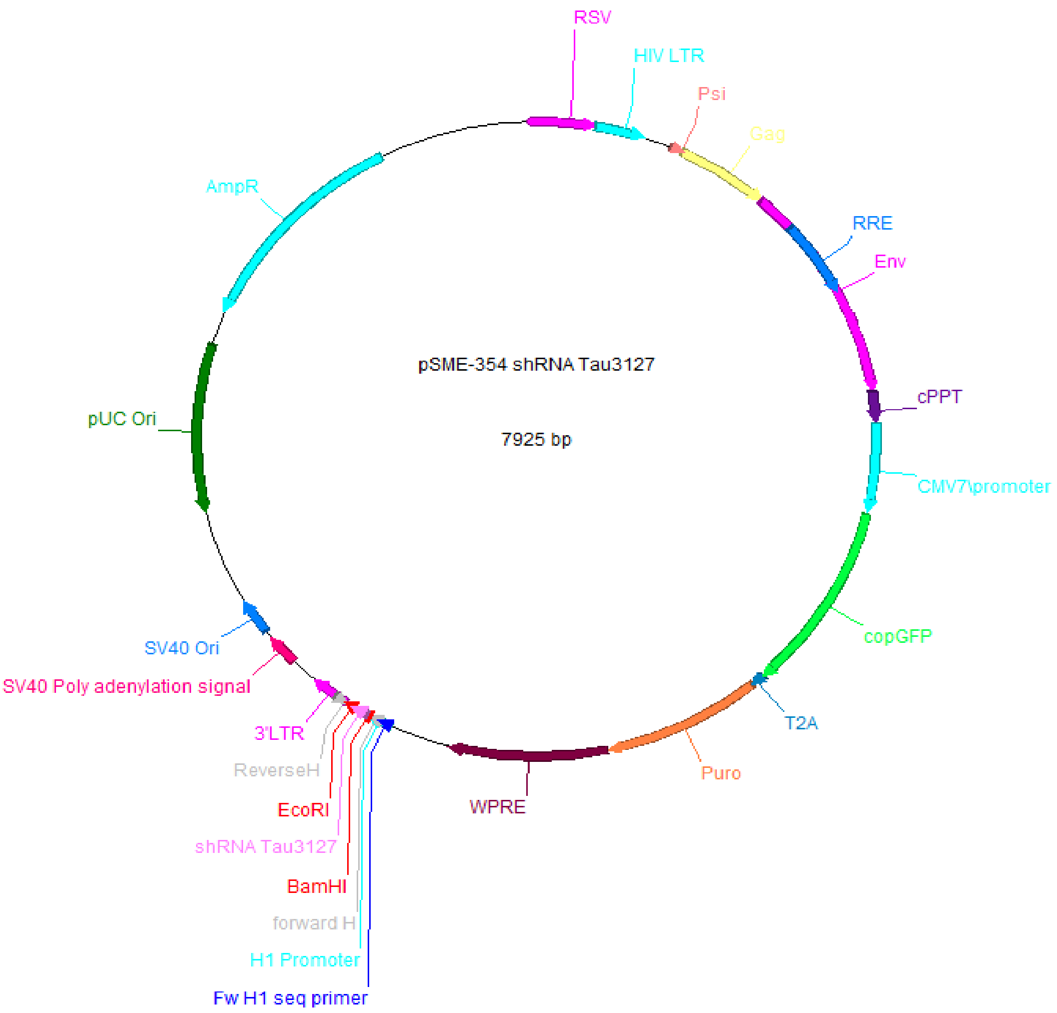

FASTA Sequence information:

>shRNA 3127 A1_H1 Promoter FWD

GGGATCTTATAAGTTCTGTATGAGACCACTTGGATCCAGCAGACGATGTCAACCTTGTGCTTCCTGTCAGACACAAGG
TTGACATCGTCTGCCTTTTTGAATTCTTCGATTCTGCTTTTTGCTTCTACTGGGTCTCTCTGGTTAGACCAGATCTGAGCC
TGGGAGCTCTCTGGCTAACTAGGGAACCCACTGCTTAAGCCTCAATAAAGCTTGCCTTGAGTGCTTCAAGTAGTGTGT
GCCCGTCTGTTGTGTGACTCTGGTAACTAGAGATCCCTCAGACCCTTTCAGTCAGTGTGGAAAATCTCTAGCAGTAGTA
GTTCATGTCATCTTATTATTCAGTATTTATAACTTGCAAAGAAATGAATATCAGAGAGTGAGAGGAACTTGTTTATTGC
AGCTTATAATGGTTACAAATAAAGCAATAGCATCACAAATTTCACAAATAAAGCATTTTTTTCACTGCATTCTAGTTGTG
GTTTGTCCAAACTCATCAATGTATCTTATCATGTCTGGCTCTAGCTATCCCGCCCCTAACTCCGCCCATCCCGCCCCTAA
CTCCGCCCAGTTCCGCCCATTCTCCGCCCCATGGCTGACTAATTTTTTTTATTTATGCAGAGGCCGAGGCCGCCTCGGCC
TCTGAGCTATTCCAGAAGTAGTGAGGAGGCTTTTTTGGAGGCCTAGACTTTTGCAGAGACCAAATTCGTAATCATGTC
ATAGCTGTTTCCTGTGTGAAATTGTTATCCGCTCACAATTCCACACAACATACGAGCCGGAAGCATAAAGTGTAAAGCC

TGGGGTGCCTAATGAGTGAGCTAACTCACATTAATTGCGTTGCGCTCACTGCCCGCTTTCCAGTCGGGAAACCTGTCG
TGCCAGCTGCATTAATGAATCGGCCAACGCGCGGGGAGAGGCGGTTTGCGTATTGGGCGCTCTTCCGCTTCCTCGCTC
ACTGACTCGCTGCGCTCGGTCGTTCGGCTGCGGCGAGCGGTATCAGCTCACTCAAAGGCGGTAATACGGTTATCCMC
AGAATCAGGGGATAACGCAGGAAAGAACATGTGAGCAAAAGGCCAGCAAAAGGCCAGGAACCGTAAAAAGGCCGC
GTTGCTGGCGTTTTTCCWAAGGCTCCGCCCCCCTGACGAGCATCMMAAAATCRACGCTCAAGTCAAAGGNGGCGAA
ACCCGA

>shRNA 3127 B1_H1 Promoter FWD

ATTTGGGATCTTATAAGTTCTGTATGAGACCACTTGGATCCAGCAGACGATGTCAACCTTGTGCTTCCTGTCAGACACA
AGGTTGACATCGTCTGCCTTTTTGAATTCTTCGATTCTGCTTTTTGCTTCTACTGGGTCTCTCTGGTTAGACCAGATCTG
AGCCTGGGAGCTCTCTGGCTAACTAGGGAACCCACTGCTTAAGCCTCAATAAAGCTTGCCTTGAGTGCTTCAAGTAGT
GTGTGCCCGTCTGTTGTGTGACTCTGGTAACTAGAGATCCCTCAGACCCTTTCAGTCAGTGTGGAAAATCTCTAGCAGT
AGTAGTTCATGTCATCTTATTATTCAGTATTTATAACTTGCAAAGAAATGAATATCAGAGAGTGAGAGGAACTTGTTTA
TTGCAGCTTATAATGGTTACAAATAAAGCAATAGCATCACAAATTTCACAAATAAAGCATTTTTTTCACTGCATTCTAGT
TGTGGTTTGTCCAAACTCATCAATGTATCTTATCATGTCTGGCTCTAGCTATCCCGCCCCTAACTCCGCCCATCCCGCCC
CTAACTCCGCCCAGTTCCGCCCATTCTCCGCCCCATGGCTGACTAATTTTTTTTATTTATGCAGAGGCCGAGGCCGCCTC
GGCCTCTGAGCTATTCCAGAAGTAGTGAGGAGGCTTTTTTGGAGGCCTAGACTTTTGCAGAGACCAAATTCGTAATCA
TGTCATAGCTGTTTCCTGTGTGAAATTGTTATCCGCTCACAATTCCACACAACATACGAGCCGGAAGCATAAAGTGTAA
AGCCTGGGGTGCCTAATGAGTGAGCTAACTCACATTAATTGCGTTGCGCTCACTGCCCGCTTTCCAGTCGGGAAACCT
GTCGTGCCAGCTGCATTAATGAATCGGCCAACGCGCGGGGAGAGGCGGTTTGCGTATTGGGCGCTCTTCCGCTTCCTC
GCTCACTGACTCGCTGCGCTCGGTCGTTCGGCTGCGGCGAGCGGTATCAGCTCACTCAAAGGCGGTAATACGGTTATC
CACAGAATCAGGGGATAACGCAGGAAAGAACATGTGAGCAAAAGGCCAGCAAAAGGCCAGGAACCGTAAAAAGGC
CGCGTTGCTGGCGTTTTTCMNAGGCTCC

pSME-358 shRNA p53:

Full map:

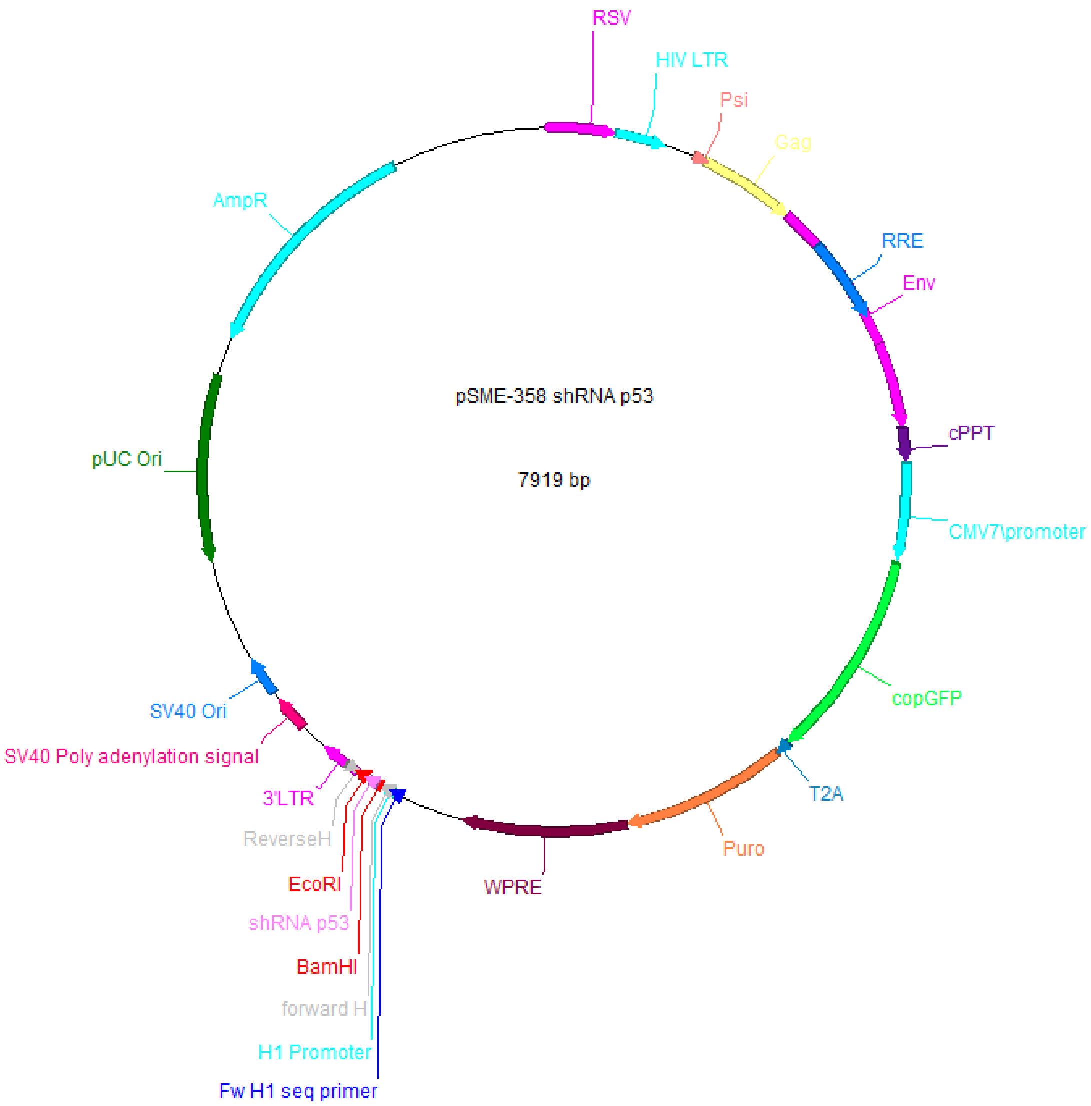

FASTA Sequence information:

>shRNA p53 A_H1 Promoter FWD

NTTGGGATCTTATAAGTTCTGTATGAGACCACTTGGATCCGACTCCAGTGGTAATCTACCTTCCTGTCAGAGTAGATTA
CCACTGGAGTCTTTTTGAATTCTTCGATTCTGCTTTTTGCTTCTACTGGGTCTCTCTGGTTAGACCAGATCTGAGCCTGG
GAGCTCTCTGGCTAACTAGGGAACCCACTGCTTAAGCCTCAATAAAGCTTGCCTTGAGTGCTTCAAGTAGTGTGTGCC
CGTCTGTTGTGTGACTCTGGTAACTAGAGATCCCTCAGACCCTTTCAGTCAGTGTGGAAAATCTCTAGCAGTAGTAGTT
CATGTCATCTTATTATTCAGTATTTATAACTTGCAAAGAAATGAATATCAGAGAGTGAGAGGAACTTGTTTATTGCAGC
TTATAATGGTTACAAATAAAGCAATAGCATCACAAATTTCACAAATAAAGCATTTTTTTCACTGCATTCTAGTTGTGGTT
TGTCCAAACTCATCAATGTATCTTATCATGTCTGGCTCTAGCTATCCCGCCCCTAACTCCGCCCATCCCGCCCCTAACTCC
GCCCAGTTCCGCCCATTCTCCGCCCCATGGCTGACTAATTTTTTTTATTTATGCAGAGGCCGAGGCCGCCTCGGCCTCT
GAGCTATTCCAGAAGTAGTGAGGAGGCTTTTTTGGAGGCCTAGACTTTTGCAGAGACCAAATTCGTAATCATGTCATA
GCTGTTTCCTGTGTGAAATTGTTATCCGCTCACAATTCCACACAACATACGAGCCGGAAGCATAAAGTGTAAAGCCTG
GGGTGCCTAATGAGTGAGCTAACTCACATTAATTGCGTTGCGCTCACTGCCCGCTTTCCAGTCGGGAAACCTGTCGTG

CCAGCTGCATTAATGAATCGGCCAACGCGCGGGGAGAGGCGGTTTGCGTATTGGGCGCTCTTCCGCTTCCTCGCTCAC
TGACTCGCTGCGCTCGGTCGTTCGGCTGCGGCGAGCGGTATCAGCTCACTCAAAGGCGGTAATACGGTTATCCACAG
AATCAGGGGATAACGCAGGAAAGAACATGTGAGCAAAAGGCCAGCAAAAGGCCAGGAACCGTAAAAAGGCCGCGT
TGCTGGCGTTTTTCCATAGGCTCCGCCCCCCTGACGAGCATCMCAAAAATCGACGCTCAAGTCAAAGGTGGCGAAAC
CCGACAGGACTWTAAAGATACCAGGCGTTTCCCCCTGGAAGCTCCCTCGKGSSCTYYCCTGTTCGAACCTGCCGCTTAC
CGGAWACCTGTTCGCCTTTYYYCCTTCGGGA

>shRNA p53 B_H1 Promoter FWD

TNTGGGATCTTATAAGTTCTGTATGAGACCACTTGGATCCGACTCCAGTGGTAATCTACCTTCCTGTCAGAGTAGATTA
CCACTGGAGTCTTTTTGAATTCTTCGATTCTGCTTTTTGCTTCTACTGGGTCTCTCTGGTTAGACCAGATCTGAGCCTGG
GAGCTCTCTGGCTAACTAGGGAACCCACTGCTTAAGCCTCAATAAAGCTTGCCTTGAGTGCTTCAAGTAGTGTGTGCC
CGTCTGTTGTGTGACTCTGGTAACTAGAGATCCCTCAGACCCTTTCAGTCAGTGTGGAAAATCTCTAGCAGTAGTAGTT
CATGTCATCTTATTATTCAGTATTTATAACTTGCAAAGAAATGAATATCAGAGAGTGAGAGGAACTTGTTTATTGCAGC
TTATAATGGTTACAAATAAAGCAATAGCATCACAAATTTCACAAATAAAGCATTTTTTTCACTGCATTCTAGTTGTGGTT
TGTCCAAACTCATCAATGTATCTTATCATGTCTGGCTCTAGCTATCCCGCCCCTAACTCCGCCCATCCCGCCCCTAACTCC
GCCCAGTTCCGCCCATTCTCCGCCCCATGGCTGACTAATTTTTTTTATTTATGCAGAGGCCGAGGCCGCCTCGGCCTCT
GAGCTATTCCAGAAGTAGTGAGGAGGCTTTTTTGGAGGCCTAGACTTTTGCAGAGACCAAATTCGTAATCATGTCATA
GCTGTTTCCTGTGTGAAATTGTTATCCGCTCACAATTCCACACAACATACGAGCCGGAAGCATAAAGTGTAAAGCCTG
GGGTGCCTAATGAGTGAGCTAACTCACATTAATTGCGTTGCGCTCACTGCCCGCTTTCCAGTCGGGAAACCTGTCGTG
CCAGCTGCATTAATGAATCGGCCAACGCGCGGGGAGAGGCGGTTTGCGTATTGGGCGCTCTTCCGCTTCCTCGCTCAC
TGACTCGCTGCGCTCGGTCGTTCGGCTGCGGCGAGCGGTATCAGCTCACTCAAAGGCGGTAATACGGTTATCCACAG
AATCAGGGGATAACGCAGGAAAGAACATGTGAGCAAAAGGCCAGCAAAAGGCCAGGAACCGTAAAAAGGCCGCGT
TGCTGGCGTTTTTCCATAGGCTCCGCCCCCCTGACGAGCATCMMAAAAATCGACGCTCAGGTCRRAGGTGGCGAAAC
CCGACAGGACTWTAAAGAWNCCAGGCGTTTCCCCCTGGAAGCTCCCTCGTGSSCTYYYCTGTTCCNACCTGCCGCTTA
CGGAANACTGTTCGCCTTTTYYCCTTCGGGAA
