# Supplementary material for: Tau affects P53 function and cell fate during the DNA damage response
Source: Commun Biol. 2020 May 19;3:245. doi: 10.1038/s42003-020-0975-4 (PMC7237658; doi:10.1038/s42003-020-0975-4)
Supplement: Supplementary file 4 — Description of Additional Supplementary Files [file 42003_2020_975_MOESM4_ESM.pdf]

Description of Additional Supplementary Files

File Name: **Supplementary Data 1**

Description: This excel document contains all primary raw data used for preparing the histogram with points in the main figures and supplementary figures, one sheet per quantification as specified

File Name: **Supplementary Data 2**

Description: This file contains all sequencing data of the CRISPR/Cas9 edited exon 1 of the *MAPT* gene encoding for the protein Tau for the amino acid sequences of the SH-SY5Y cell lines shown in Figure 1, as well as the plasmid maps and their nucleotide sequence of all shRNA plasmids (Tau and p53)
