# Supplementary material for: Tau affects P53 function and cell fate during the DNA damage response
Source: Commun Biol. 2020 May 19;3:245. doi: 10.1038/s42003-020-0975-4 (PMC7237658; doi:10.1038/s42003-020-0975-4)
Supplement: Supplementary file 6 — Reporting Summary [file 42003_2020_975_MOESM6_ESM.pdf]

nature research

Corresponding author(s): Paolo Paganetti
Last updated by author(s): 20/04/2020

# Reporting Summary

Nature Research wishes to improve the reproducibility of the work that we publish. This form provides structure for consistency and transparency in reporting. For further information on Nature Research policies, see Authors & Referees and the Editorial Policy Checklist.

## Statistics

For all statistical analyses, confirm that the following items are present in the figure legend, table legend, main text, or Methods section.

| n/a | Confirmed | |
|---|---|---|
| ☐ | ☒ | The exact sample size (*n*) for each experimental group/condition, given as a discrete number and unit of measurement |
| ☐ | ☒ | A statement on whether measurements were taken from distinct samples or whether the same sample was measured repeatedly |
| ☐ | ☒ | The statistical test(s) used AND whether they are one- or two-sided<br>*Only common tests should be described solely by name; describe more complex techniques in the Methods section.* |
| ☐ | ☒ | A description of all covariates tested |
| ☐ | ☒ | A description of any assumptions or corrections, such as tests of normality and adjustment for multiple comparisons |
| ☐ | ☒ | A full description of the statistical parameters including central tendency (e.g. means) or other basic estimates (e.g. regression coefficient) AND variation (e.g. standard deviation) or associated estimates of uncertainty (e.g. confidence intervals) |
| ☐ | ☒ | For null hypothesis testing, the test statistic (e.g. *F*, *t*, *r*) with confidence intervals, effect sizes, degrees of freedom and *P* value noted<br>*Give P values as exact values whenever suitable.* |
| ☒ | ☐ | For Bayesian analysis, information on the choice of priors and Markov chain Monte Carlo settings |
| ☒ | ☐ | For hierarchical and complex designs, identification of the appropriate level for tests and full reporting of outcomes |
| ☐ | ☒ | Estimates of effect sizes (e.g. Cohen's *d*, Pearson's *r*), indicating how they were calculated |

*Our web collection on statistics for biologists contains articles on many of the points above.*

## Software and code

Policy information about availability of computer code

| | |
|---|---|
| Data collection | Nikon C2 microscope license was used to collect all the immunofluorescence and comet data, Licor Odyssey CLx was used for immunoblot and in-cell western. LionHeart FX, BioTek to capture B-Galactosidase images. Tecan Infinite M Plex was used for MTS and LDH assay. Biorad CFX Connect real-time system for the qPCR. |
| Data analysis | Image Studio ver5 Licor was used to analysed the immunoblot data, ImageJ was used for immunofluorescence quantification. Microsoft Excel was used for senescence and qPCR analyses. CaspLab was used for comet assay.<br>GraphPad Prism version 7.02 and IBM SPSS Statistics V20 was used to perform all the statistical analyses. |

For manuscripts utilizing custom algorithms or software that are central to the research but not yet described in published literature, software must be made available to editors/reviewers. We strongly encourage code deposition in a community repository (e.g. GitHub). See the Nature Research guidelines for submitting code & software for further information.

## Data

Policy information about availability of data

All manuscripts must include a data availability statement. This statement should provide the following information, where applicable:

- Accession codes, unique identifiers, or web links for publicly available datasets
- A list of figures that have associated raw data
- A description of any restrictions on data availability

The raw data for all the figures and supplementary figures are included as a supplementary data file. All genomic sequencing data generated by the external provider (Microsynth AG, Balgach, Switzerland) for the CRISPR/Cas9 edited exon of MAPT, and all maps and sequences of the gene-editing plasmids and shRNA plasmids are included as supplementary material. All the data generated and/or analyzed, all plasmids and cell lines included in the current study are available from the corresponding authors on reasonable request

# Field-specific reporting

Please select the one below that is the best fit for your research. If you are not sure, read the appropriate sections before making your selection.

☒ Life sciences ☐ Behavioural & social sciences ☐ Ecological, evolutionary & environmental sciences

For a reference copy of the document with all sections, see nature.com/documents/nr-reporting-summary-flat.pdf

# Life sciences study design

All studies must disclose on these points even when the disclosure is negative.

| | |
|---|---|
| Sample size | Each experiment was performed at least independently three times with the exception of the qPCR analysis where few transcripts were analyzed in a single experiment using technical triplicates and comparing two independent cell lines. Sample sizes and number of repeats are defined in each figure legends. Samples size was determined based on optimization studies. |
| Data exclusions | All experiments were performed with cells maintained for a maximum of one month in culture, therefore data generated with older cultures were excluded from this work. |
| Replication | All results were replicated with small variation in the extent of the effects, therefore representative results were included. |
| Randomization | No animal and human research was used and any randomization was performed. |
| Blinding | Whenever possible, data collection and data analysis were performed by different individuals, and key experiments were conducted by different individuals. |

# Reporting for specific materials, systems and methods

We require information from authors about some types of materials, experimental systems and methods used in many studies. Here, indicate whether each material, system or method listed is relevant to your study. If you are not sure if a list item applies to your research, read the appropriate section before selecting a response.

## Materials & experimental systems

| n/a | Involved in the study |
|---|---|
| ☐ | ☒ Antibodies |
| ☐ | ☒ Eukaryotic cell lines |
| ☒ | ☐ Palaeontology |
| ☒ | ☐ Animals and other organisms |
| ☒ | ☐ Human research participants |
| ☒ | ☐ Clinical data |

## Methods

| n/a | Involved in the study |
|---|---|
| ☒ | ☐ ChIP-seq |
| ☒ | ☐ Flow cytometry |
| ☒ | ☐ MRI-based neuroimaging |

## Antibodies

| | |
|---|---|
| Antibodies used | Tau13 (sc-21796, Santa Cruz), pS129-H2A.X (sc-517348, Santa Cruz), pS1981-ATM (#13050, Cell Signaling), pT68-Chk2 (#2197, Cell Signaling), pS428-ATR (#2853, Cell Signaling), P53 (sc-126, SantaCruz,), pS15-P53 (ab223868, Abcam), MDM2 (#86934, Cell Signaling), ClAsp175-Caspase3 (#9661, Cell Signaling) p21 (SC-53870), secondary antibodies anti-mouse IgG-Alexa594 or -Alexa 488 (A-11032, A-11001, ThermoFisher Scientific) or anti-rabbit IgG-Alexa594 or -Alexa488 (A-11037, A-11034, ThermoFisher Scientific), DAPI (D9542, Sigma-Aldrich), GAPDH (ab181602, Abcam), P53 (#2524, Cell signaling), biotinylated HT7 antibody (MN1000B, ThermoFisher Scientific), streptavidin-IRDye (926-32230, Licor Biosciences), Vista Green DNA Dye (#235003, Cell Biolabs).<br>For immunostaining analyses, antibodies were diluted in a 1:500 ratio, except for Tau13 (1:200 dilution), ClAsp175-Caspase3 (1:1,000) and the secondary antiboied (used in a 1:1,000 ratio). For immunoblotting analyses all antibodies are used in a dilution 1:1,000 except for GAPDH and secondary antibodies (1:5,000 dilution). For Comet Assay the dye was diluted in a 1:10,000 ratio. |
| Validation | Tau13 antibody was validated using Knocked-out cells for Tau both by immuno staining and western blot. The commercial antibodies used in this study were validated by the manufacturer. The dilution of each antibody was used based on a thorough prior investigation both in our labs and in other labs. |

## Eukaryotic cell lines

Policy information about cell lines

| | |
|---|---|
| Cell line source(s) | SH-SY5Y cells were purchased from Sigma-Aldrich, code 94030304 and maintained in DMEM supplemented with 10% fetal bovine serum, 1% non-essential amino acids, 1% penicillin-streptomycin (Gibco). IMR5, IMR32 and SK-N-AS were purchased |

by Chiara Brignole from IRCCS Istituto Giannina Gaslini maintained in DMEM supplemented with 10% fetal bovine serum, 1% non-essential amino acids, 1% penicillin-streptomycin (Gibco).
In our lab were then generated SH-SY5Y,IMR5 and IM32 cells Knocked-out and Knocked-down for Tau as described in the section of the paper materials and methods. For the tansfections HEK-293 cells were obtained from System Biosciences and maintained in complete media, DMEM supplemented with 10% fetal bovine serum, 1% non-essential amino acids, 1% penicillin-streptomycin (Gibco).

| | |
|---|---|
| Authentication | Gene knockouts and knockdown were confirmed by western blot, immuno staining, immuno precipitation and sequencing. |
| Mycoplasma contamination | Cells were tested for mycoplasma once a year and they were mycoplasma negative. |
| Commonly misidentified lines (See ICLAC register) | The cell lines used in this study are not listed in the database of commonly misidentified cell lines maintained by ICLAC. |
